# Coprecipitated Silica Controls the Divergent Pathways of Cysteine-Induced Ferrihydrite Transformation

**DOI:** 10.1021/acs.est.6c02208

**Published:** 2026-08-07

**Authors:** Yinyin Zheng, Libor Kovarik, Ravi K. Kukkadapu, Qian Zhao, Maya Engel

**Affiliations:** † Department of Soil and Water Sciences, The Robert H. Smith Faculty of Agriculture, Food and Environment, The Hebrew University of Jerusalem, Rehovot 7610001, Israel; ‡ Environmental Molecular Sciences Laboratory, 6865 Pacific Northwest National Laboratory, Richland, Washington 99354, United States; § Department of Sustainable Earth Systems Sciences, 12335 The University of Texas at Dallas, Richardson, Texas 75080, United States

**Keywords:** iron mineral, dissolution−precipitation, carbon stabilization, biogeochemical cycling

## Abstract

The reductive transformation of ferrihydrite (Fh) is a key process controlling iron mineral evolution and mineral-associated organic matter (MAOM) cycling in soils and sediments, yet the regulating role of coprecipitated silica remains poorly constrained. Here, we investigated the influence of coprecipitated silica on cysteine-induced Fh transformation under anoxic conditions using Fh and Si-Fh coprecipitates with Si/Fe molar ratios of 0.05 and 0.1. Mineral evolution and Fe speciation were monitored using X-ray diffraction (XRD), Mössbauer spectroscopy, X-ray photoelectron spectroscopy (XPS), transmission electron microscopy coupled with energy-dispersive X-ray analysis (TEM-EDX), and selective chemical extractions. In silica-free Fh, cysteine induced rapid transformation into lepidocrocite through reductive dissolution and recrystallization pathways that largely excluded silica and MAOM from the newly formed mineral phase. In contrast, coprecipitated silica altered the extent, rate, and pathway of mineral evolution. At a Si/Fe ratio of 0.05, crystalline phase formation was delayed and redirected toward a porous-like goethite phase that retained substantial silica and organic associations. At a Si/Fe ratio of 0.1, formation of crystalline secondary phases was strongly suppressed, coinciding with accumulation of labile surface-associated Fe­(III). These findings demonstrate that coprecipitated silica can decouple reductive electron transfer from mineral recrystallization and alter the fate of MAOM during Fh transformation under anoxic conditions.

## Graphical abstract

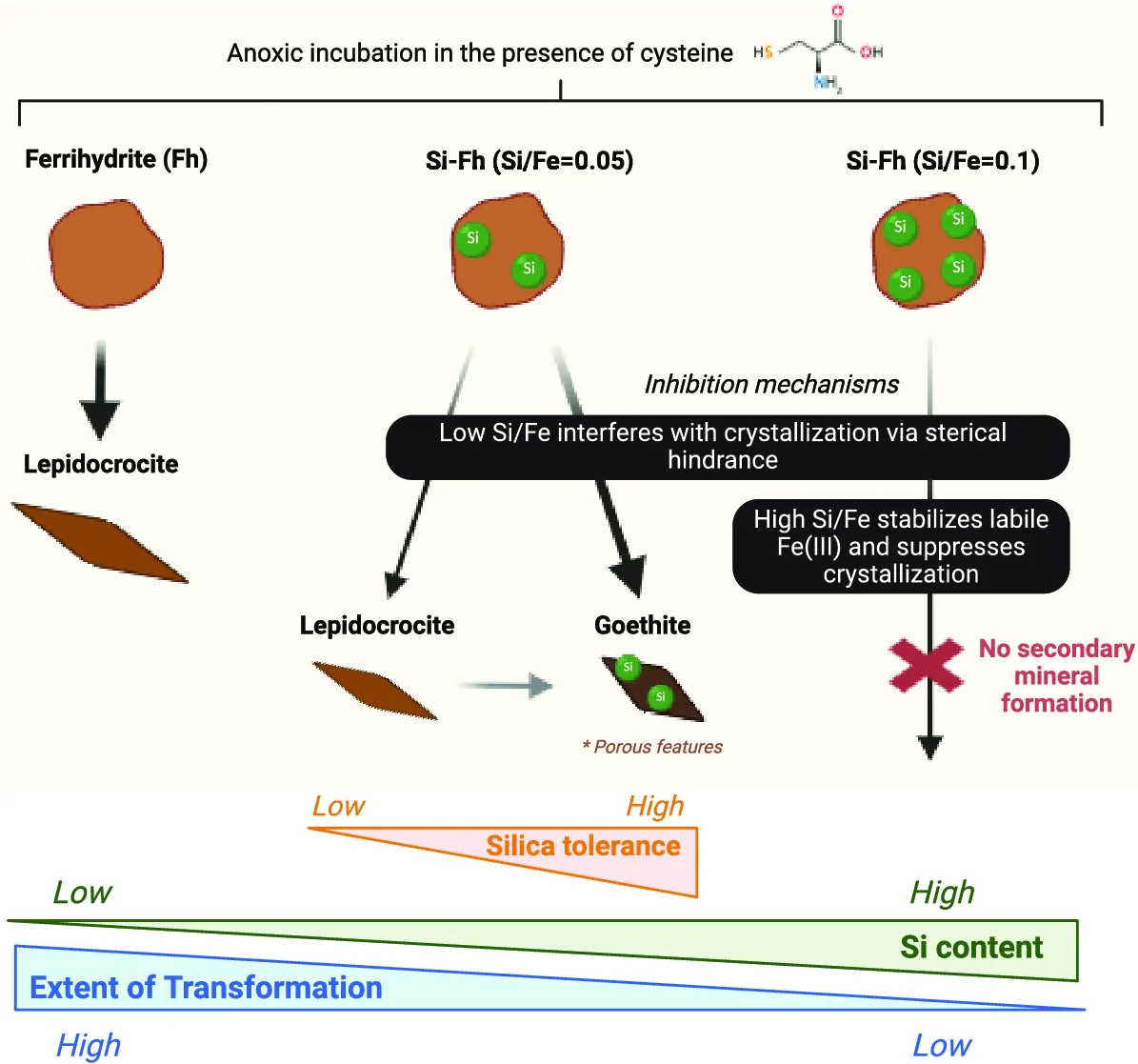

## Introduction

Ferrihydrite (Fh) is a nanocrystalline Fe­(III) oxyhydroxide, ubiquitous in soils and sediments.^1^ Due to its high surface area and reactivity, Fh readily associates with inorganic and organic compounds. Oxyanions such as phosphate and silicate, together with dissolved organic matter (OM), commonly bind to and coat Fh surfaces.^2–7^ Silicate anions limit Fe–O–Fe polymerization by binding to Fh at Fe–O corner sites via inner-sphere Si–O–Fe bonds.^8^ Extended X-ray absorption fine structure (EXAFS) and X-ray photoelectron spectroscopy (XPS) analyses indicate that disruption of corner-sharing Fe­(III) octahedra results in a more amorphous mineral structure composed of smaller and more disordered crystallites.^7,9^

Under anoxic and ferruginous conditions, dissolved Fe­(II) can bind to Fh and catalyze its transformation into more crystalline phases such as lepidocrocite, goethite, or magnetite, depending on factors like solution pH.^10–13^ Recent studies further show that the transformation rate and product distribution depend on the initial crystallinity of Fh and the identity of coexisting Fe mineral phases.^14,15^ These Fe phase transformations play an integral role in soil biogeochemical cycling and may influence the speciation and mobility of co-occurring elements. OM coatings of the Fh, known as mineral-associated OM (MAOM), can physically block reactive sites on the Fh surface, influencing the extent and kinetics of its Fe­(II)-catalyzed transformation.^16,17^ However, not all organic coatings passivate or stabilize the mineral surface-redox; active organic functionalities such as hydroquinones or thiols, can abiotically reduce structural Fe­(III) in Fh.^18–20^ For example, upon binding of the amino acid cysteine to Fh under anoxic conditions, thiol-driven reductive dissolution of the Fh facilitates its transformation into more crystalline phases.^18,19,21,22^

Similar to the inhibitory effects of organic coatings, even low levels of coprecipitated silica can inhibit Fe­(II)-catalyzed Fh transformation, as evidenced by decreased Fe atom exchange and suppressed nucleation of secondary minerals in Si-Fh coprecipitates upon exposure to dissolved Fe­(II).^7^ However, these studies introduced the Fe­(II) into the solution containing the Si-Fh minerals, and did not consider Fe­(II) that is produced on the mineral surface—*e.g.*, as induced by certain redox-active organic ligands.^18,19^ This difference in the initial source of the Fe­(II) is likely to impact the distribution of Fe­(II) and Fe­(III) species and their partitioning between the aqueous and solid-mineral phase, and in turn, may modify Fh transformation dynamics.

Although the effects of OM and silica (either adsorbed or coprecipitated) on Fh reactivity have been studied independently,^6,8,23,24^ their combined effects on the extent, kinetics, and mechanisms of Fh transformation remain poorly constrained.^25^ A recent floodplain groundwater study revealed that silica-organic coatings of Fh nanocolloids passivate the mineral surface, allowing them to persist under anoxic and ferruginous conditions, suggesting Si-OM coassociations as a mineral stabilizing mechanism in situ.^26^ Yet, it remains unclear how Si-OM-Fh interactions influence mineral transformation dynamics and the fate of MAOM in continuously reducing and organic matter-rich environments.^22,27^

Here, we monitored the temporal transformation dynamics of Fh and Si-Fh coprecipitates with Si/Fe ratios of 0.05 and 0.1 during cysteine-mediated transformation under anoxic conditions. Cysteine was selected as a model redox-active ligand because thiol-containing compounds are common components of natural OM and microbial metabolites in reducing environments such as wetlands, peat soils, rice paddies, sediments, and anoxic porewaters.^28,29^ Using a combination of X-ray diffraction (XRD), XPS, Mössbauer spectroscopy, transmission electron microscopy (TEM) coupled with energy-dispersive X-ray (EDX) analysis, and select chemical extractions, we characterize the structural features of the transformation products, as well as the extent and spatial distribution of silica and MAOM. We show that silica-OM coassociations not only delay Fh transformation, but also modify transformation pathways, alter nanoscale characteristics of newly formed Fe phases, and spatially segregate silica and MAOM distributions.

## Materials and Methods

### Synthesis of Fh and Si-Fh Coprecipitates

Two-line Fh and Si-Fh coprecipitates were synthesized following a coprecipitation method^30^ (all syntheses were conducted in plastic beakers). For Fh, a 0.20 M ferric nitrate (Fe­(NO_3_)_3_·9H_2_O) solution was titrated with 1 M KOH under vigorous stirring until a brown precipitate formed at pH ∼ 7. The solid products were washed via consecutive centrifugation cycles (24,149*g*, 30 min) until the conductivity of the supernatant equaled that of deionized water, then freeze-dried (−20 °C) as powders and stored in the dark at room temperature. To produce Si-Fh, Na_2_SiO_3_ was added into the 1 M KOH solution at either 0.033 or 0.066 M,^31,32^ yielding bulk Si/Fe ratios of 0.05 and 0.1, respectively (see Table S1 for details), hereafter referred to as Si-Fh_0.05 and Si-Fh_0.1. The Si/Fe ratios were selected to represent environmentally relevant low and moderate Si enrichments reported for natural siliceous-Fh and Fe oxide-rich soils and sediments.^33^ Mineral identification and purity were confirmed by TEM and XRD (see details below).

### Mineral Incubations in the Presence of Cysteine

The Fh and Si-Fh minerals were exposed to cysteine in batch experiments under anoxic conditions, maintained by performing all experimental steps inside a Coy Laboratories anaerobic chamber (98:2 N_2_:H_2_). Triplicate samples were set up for each mineral by introducing 25 mg into 20 mL of a ∼1100 μM cysteine solution prepared in 0.01 M NaCl at pH 7. This cysteine concentration was selected to reflect high levels of redox-active OM that may exist in anoxic soils and sediments.^19,34^ Control samples were prepared in the absence of minerals. All samples were then incubated in the dark, at room temperature, and under constant agitation of 250 rpm, for different time periods ranging from 5 min to 25 days. At the designated incubation time, an aliquot of the suspension was filtered through a 0.45 μm PES syringe filter and the filtrate was collected for aqueous-phase analysis. For the solid-phase analysis, the remaining solution was filtered through a 0.45 μm PES filter paper using vacuum filtration. The filtered mineral samples were then dried inside the anaerobic chamber and stored in the dark in glass serum vials with butyl rubber stoppers to prevent oxidation.

### Aqueous-Phase Analysis

Filtered solutions were immediately analyzed for dissolved Fe­(II) concentrations using the ferrozine assay,^35^ with absorbance measured at 562 nm on a BioTek Synergy H1 microplate reader. Cysteine and cystine levels were quantified via fluorenylmethyloxycarbonyl chloride (FMOC-Cl) derivatization followed by high-performance liquid chromatography (HPLC; Vanquish system, Thermo Fisher Scientific)^36^ with UV detection at 265 nm (see Section S1 in the Supporting Information (SI) for details). pH was monitored in all samples, and aqueous S, Si, and Fe concentrations were measured using inductively coupled plasma optical emission spectroscopy (ICP-OES; iCAP PRO XP Duo, Thermo Scientific).

### Solid-Phase Analysis

The mineral phase was first characterized using XRD to identify mineral transformation products. Measurements were conducted on a Bruker D8 Advance diffractometer equipped with a LYNXEYE-XE-T detector using Cu Kα radiation (λ = 1.5418 Å), operated at 40 kV and 40 mA. Mineral powder samples were mounted on low-background quartz holders and scanned over a 2θ range of 10–80° with a step size of 0.02° and a counting time of 0.5 s per step. Although measurements were not conducted under anoxia, the mineral phase distribution was not expected to have changed due to the dry conditions and short exposure time. The proportions of each mineral phase were quantified through Rietveld refinement of the diffraction patterns using the TOPAS software (see Section S2 and Figure S1 for details).^6,37–39^ In brief, crystalline phases were modeled using published crystallographic structures and the remaining fraction was attributed to the poorly crystalline/amorphous Fh or Si-Fh phase.

Structure and spatial composition of the minerals were further evaluated at the nanoscale using TEM-EDX analysis with a set of Thermo Fisher Scientific microscopes (see Section S3 for details). XPS analysis was utilized to measure the elemental composition on the mineral surface (see Section S4 for details), and Fourier-transform infrared spectroscopy (FTIR) was used to assess chemical bonding and functional group composition (see Section S5 for details).

Mössbauer spectra of the filtered mineral samples were collected at multiple temperatures (298 K, 80 K, 77 K, 60 K, 12 K) at the Environmental Molecular Sciences Laboratory (EMSL) in Washington, USA, using either a Web Research Company instrument (St. Paul, MN, USA) or a WissEl Elektronik unit (Germany), to identify the iron mineral composition. Sample encapsulation into Mössbauer disks was carried out in an anaerobic chamber maintained at <1 ppm of O_2_. The disks were sealed with aluminized Mylar film and rapidly transferred into the Mössbauer instrument to minimize exposure to atmospheric oxygen. These setups included a closed-cycle cryostat (SHI-850, Janis Research Company, Inc., Wilmington, MA, USA), a Sumitomo CKW-21 helium compressor unit (Allentown, PA, USA), and a Ritverc NaI detection system for the Web Research unit, or an Ar–Kr proportional counter detector for the WissEl system. A ^57^Co/Rh source was used as the γ energy source. Data acquisition and processing followed previously reported procedures^40^ (see Section S6 for details).

### Chemical Extractions

Chemical extractions were performed under anoxic conditions to quantify surface-associated Fe­(II) and Fe­(III) species.^21,22,41^ For the labile surface-associated Fe­(III), samples from select incubation time points were centrifuged (10,733*g*, 30 min) to separate the solid and aqueous phase. Following centrifugation, 12.3 mL of the supernatant was carefully removed, and 300 μL of 5 mM xylenol orange (XO) was added into the remaining 2.7 mL suspension. The suspension was immediately vortexed for 20 s and filtered through a 0.45 μm PES syringe filter, after which 20 μL of the filtrate was diluted with 180 μL of 40 mM HCl and incubated in the dark for 15 min to allow full color development. Absorbance was then measured at 560 nm on a plate reader, and the Fe­(III) concentrations were determined using a calibration curve prepared under the same conditions.

For surface-associated Fe­(II), samples from select incubation time points were centrifuged at 10,733*g* for 30 min. The supernatant was removed and analyzed for dissolved Fe­(II) levels using the ferrozine assay. Thereafter, 15 mL of 5 mM HCl was added to the pelleted solid and shaken for 10 min to extract surface-associated Fe­(II). The suspension was then centrifuged (10,733*g*, 30 min), and the Fe­(II) concentration in the acidic supernatant was measured using the ferrozine assay. This acid extraction was repeated until Fe­(II) concentrations in the final acidic extract decreased to a low residual level, typically ∼20–50 μM, which required 2–4 repetitions. Total extracted Fe­(II) was then calculated from the sum of all acid extraction steps. The concentrations of cysteine and cystine were also measured by HPLC in the first supernatant and in each extraction step. Control experiments with Fh and Si-Fh minerals (without cysteine) confirmed that both extractions measured solely surface-associated Fe species—only trace amounts of Fe­(II) (<10 μM after 40 min contact) were released in the 5 mM HCl extraction, indicating negligible mineral dissolution, while no Fe­(III) was detected in the XO extraction. Additional solution controls containing 250 μM Fe­(III) showed 91–94% Fe­(III) recovery in the presence of cystine, Fe­(II), and both, indicating only minor interference with the XO extraction.

## Results and Discussion

### Temporal Aqueous-Phase Dynamics

Anoxic incubation of Fh and Si-Fh minerals with cysteine led to a rise in dissolved Fe­(II) (Fe­(II)_aq_) concentrations and a concurrent decline in cysteine concentrations (Table S5). Notably, while cysteine remained stable in mineral-free controls over 24 h (Table S2), it was completely consumed in the mineral treatments within the same time frame. This confirms that spontaneous cysteine oxidation is negligible and that its depletion is driven exclusively by association with the mineral phase. Simultaneously increasing cystine concentrations (Figure 1), which stabilized at ∼200 μM, demonstrate that cysteine oxidizes to cystine upon binding to Fh, which is abiotically reduced to Fe­(II) (Figure S2). The rise in Fe­(II)_aq_ levels was rapid at first, but gradually stabilized over time, consistent with the complete consumption of cysteine.

**Figure 1:**
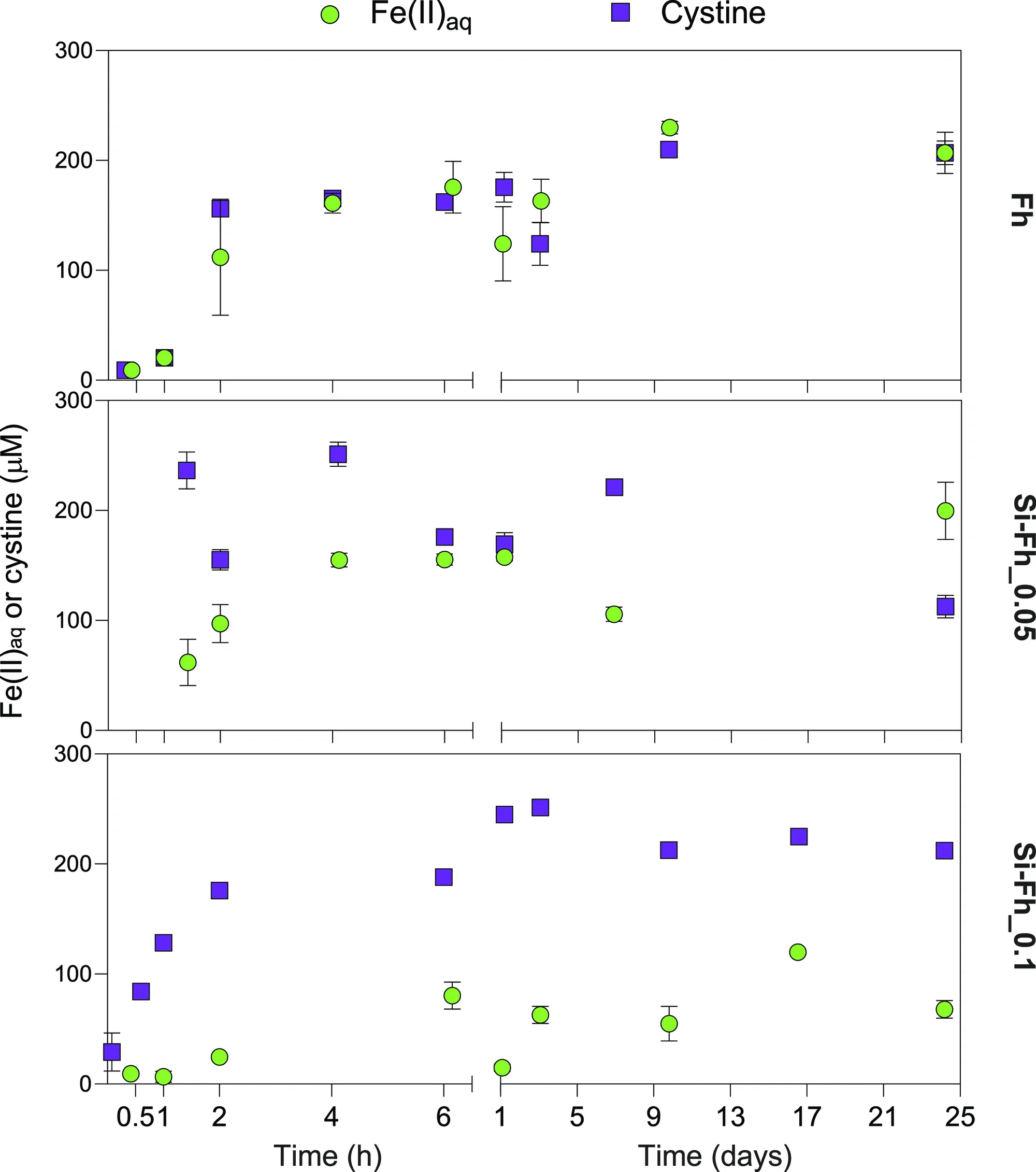
Concentrations of dissolved Fe­(II) (Fe­(II)_aq_) and cystine, following incubation with ferrihydrite (Fh; top) and Si-Fh at different Si/Fe ratios (Si-Fh_0.05; middle and Si-Fh_0.1; bottom) under anoxic conditions. Data are presented as averages with respective standard errors for 3 or 6 replicates.

Similar cysteine and cystine patterns across the Fh and Si-Fh minerals suggest that the coprecipitated silica did not influence cysteine reactivity. However, Fe­(II)_aq_ concentrations were dependent on the mineral Si/Fe ratio, with higher levels detected in the presence of Fh compared to the Si-Fh coprecipitates (Figure 1). The maximum observed concentrations were approximately 230 μM, 200 μM, and 120 μM Fe­(II)_aq_ for Fh, Si-Fh_0.05, and Si-Fh_0.1, respectively, suggesting that the coprecipitated silica regulates the dissolution dynamics of Fe­(II) following its generation (Figures S2–S3). Moreover, with observed Fe­(II)_aq_ levels of <230 μM being far below their expected values of ∼1100 μM (calculated based on the complete disappearance of cysteine; Table S5), we propose that a substantial fraction of the produced Fe­(II) occurs as surface-associated Fe­(II),^42,43^ labile, surface-associated Fe­(III), or as structural Fe in newly formed Fe phases (discussed below).

Inversely proportional to the levels of Fe­(II)_aq_ were the concentrations of dissolved Si, with approximately 20 μM and 35 μM Si released from Si-Fh_0.05 and Si-Fh_0.1, respectively (Figure S4). Previous studies have shown that silica coatings develop from the adsorption of dissolved silicate to the Fh mineral surface that can delay or inhibit Fe­(II)_aq_ adsorption^44^ and its atom exchange with structural Fe­(III) in Fh.^25^ In the presence of the Si-Fh minerals, we observed a lower net accumulation of Fe­(II)_aq_, which is typically associated with lowering the solution pH.^45^ This, combined with the presence of aqueous Si, decreased the extent of solution acidification, resulting in a ∼0.6 pH unit increase, from ∼7.0 with Fh up to ∼7.6 with Si-Fh_0.1 (Figure S4). Because the pH was below 8, Fe­(II) predominantly remains in dissolved form with limited hydrolysis, thereby restricting Fe­(OH)_2_ precipitation,^46^ and the formation of Fe–Si layers.^47^ We propose that the decrease in Fe­(II)_aq_ with increasing Si/Fe ratios reflects shifts in Fe­(II) partitioning between aqueous and surface-associated pools. Next, we examined whether these aqueous-phase differences were accompanied by divergent Fh mineral transformation pathways.

### Mineral Transformation Dynamics

Diffractograms of the incubation products of Fh (Si/Fe = 0) display how it gradually transforms into lepidocrocite, consistent with prior observations that lepidocrocite is the favored Fe­(III) oxide product when Fe­(II) is abundant and catalysis is relatively rapid^7,48^ (Figure 2 and Table S3). Transformation progressed within the first 3 days and continued throughout the incubation, with lepidocrocite identified as the dominant secondary phase. In contrast, the transformation of Si-Fh_0.1 into secondary crystalline minerals was inhibited during the 17-day incubation, in accordance with similar studies that observed retarded Fe­(II)-catalyzed transformation of Fh^7^ in the presence of silica, even when conducted at elevated temperatures.^23^ Nonetheless, we cannot exclude the possibility that longer incubation times for Si-Fh_0.1 are required for the formation of more crystalline phases.

**Figure 2:**
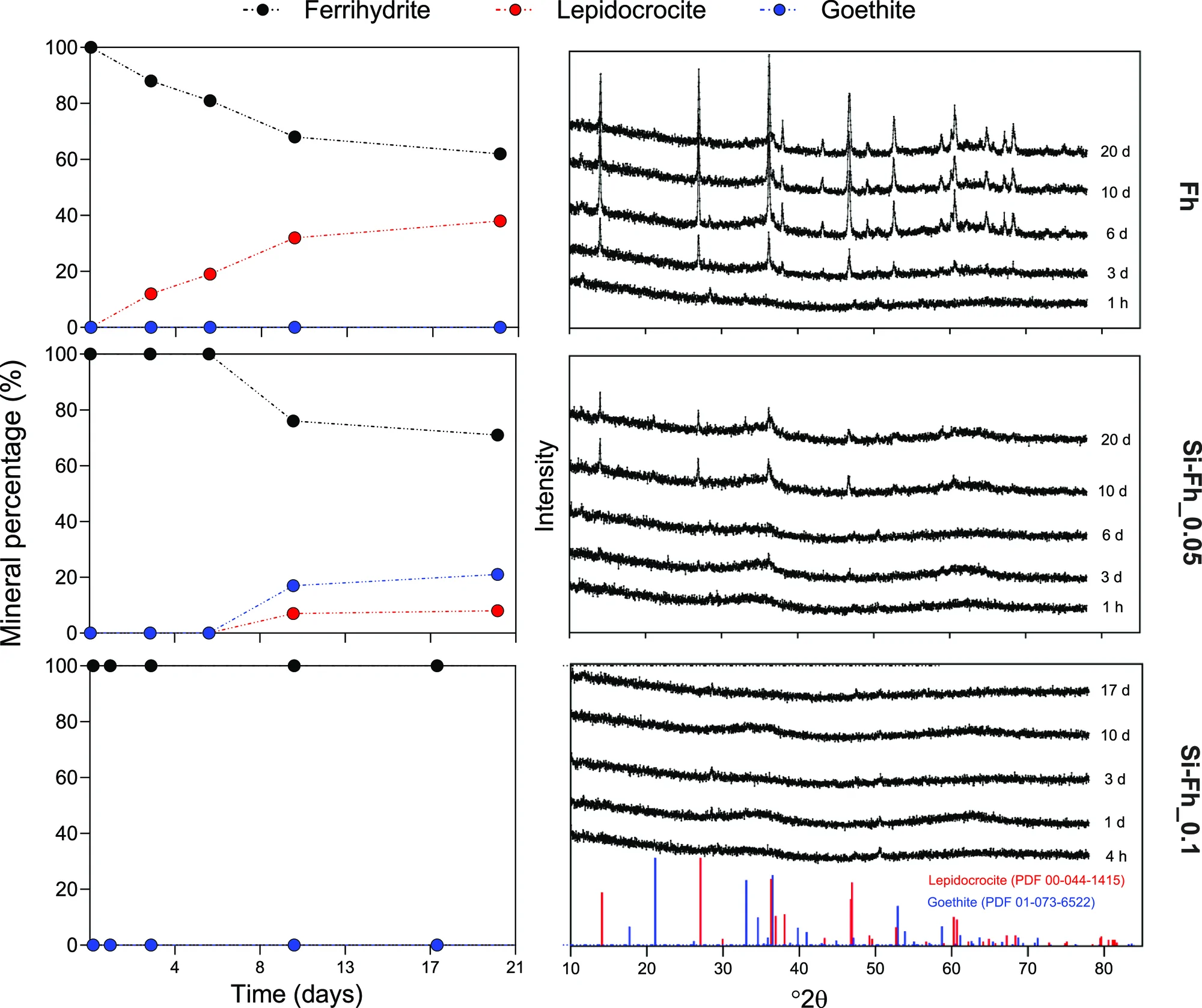
Mineral phase distribution (left) and corresponding diffractograms (right) following incubation of ferrihydrite (Fh), Si-Fh_0.05, and Si-Fh_0.1 in the presence of cysteine under anoxic conditions. Reference peaks of lepidocrocite (PDF 00–044–1415) and goethite (PDF 01–073–6522) are shown for comparison at the bottom of the Si-Fh_0.1 diffractogram panel.

The transformation dynamics of Si-Fh_0.05 differed in rate, extent, and pathway compared to both the Fh and the Si-Fh_0.1 minerals (Figure 2). While silica loading in Si-Fh_0.05 was insufficient to inhibit crystallization, it delayed its onset and decreased the extent of secondary mineral formation compared to Fh. With the appearance of crystalline phases after 10 days, two distinct phases were identified—lepidocrocite and goethite—with the latter estimated to contribute more substantially (Table S3). This indicates that the lower silica loading not only delays Fh transformation into more crystalline phases but can also direct the crystallization pathway.

The Mössbauer spectra of the untreated Fh and Si-Fh_0.05 collected at multiple temperatures (Figure S5) and their fitted parameters at 12 and 60 K (Table S4) show close agreement with those reported at 4.2 K by Murad et al.^49^ and at 65 K by Chen et al.^3^ for laboratory-synthesized 2-line Fh. The spectrum of the untreated Fh at 60 K appears as a collapsed sextet (Figure 3), unlike the mineral mixture obtained after Fh’s 6-day anoxic incubation with cysteine that displayed clear sextet features. This mixture was best fitted by Fh and lepidocrocite, with minor features attributed to goethite (Figure 3, bottom left). The percentage of lepidocrocite quantified through Mössbauer spectra fitting (∼14%; Table S4) was similar to that estimated from XRD (∼19%; Table S3), with both analyses confirming lepidocrocite as the major transformation product of Fh after 6 days.

**Figure 3:**
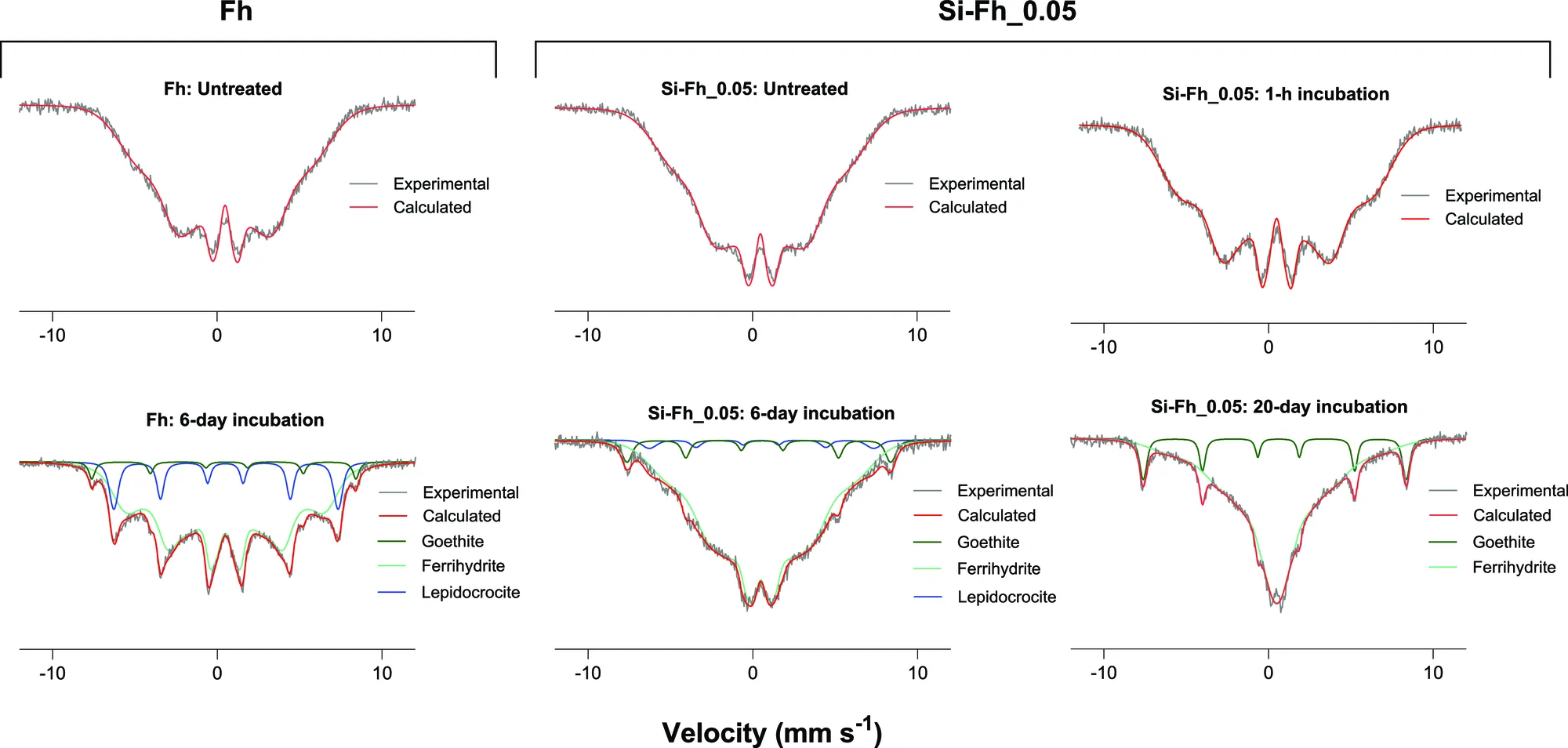
Mössbauer spectra collected at 60 K for ferrihydrite (Fh; left) and Si-Fh_0.05 (right) before (i.e., untreated) and after incubation with cysteine under anoxic conditions. Experimental data (gray) and calculated spectra (red) are shown, with spectral components assigned to Fh, goethite, and lepidocrocite as indicated. Incubation times are 1 h, 6 days, and 20 days.

Following the 6-day anoxic incubation of Si-Fh_0.05, the mineral mixture exhibited Mössbauer characteristics that differed significantly from its Fh analog, in both the 60 K (Figure 3) and 12 K spectra (Figure S6). Specifically, this cysteine-treated Si-Fh sample lacked well-defined lepidocrocite sextet features (both at 60 and 12 K), revealed small amounts of goethite (Table S4), and presented greater magnetic ordering of the residual Fh that is commonly associated with increased crystallinity.^50,51^ This supports the notion that a bulk amount of the residual Fh is either partially recrystallized or structurally modified, highlighting that the Fh and Si-Fh_0.05 are actively cycled, with a relatively more crystalline Fh product than its precursor Fh phase. At Si-Fh_0.05′s longer incubation time of 20 days, goethite was still identified as the dominant transformation product by both Mössbauer (∼12%; Table S4) and XRD (∼21%; Table S3) analyses.

The discrepancies between XRD and Mössbauer results likely arise from the different sensitivities of these techniques to poorly crystalline, nanocrystalline, and minor mineral phases, as well as the uncertainties associated with the fitting results. Accordingly, we emphasize the temporal trends in Fh and Si-Fh transformation dynamics, and consider the reported mineral proportions to be semiquantitative rather than exact. Together, the results of the XRD and Mössbauer analyses demonstrate that coprecipitated silica modifies the Fh transformation pathway, resulting in less transformation into crystalline minerals, alongside a preference toward the evolution of goethite instead of lepidocrocite. To investigate the mechanisms responsible for these observations, we quantified Fe­(II) and Fe­(III) species distribution in aqueous and surface-associated pools during the Fh and Si-Fh mineral incubations—described below.

### Distribution of Fe Species: Dissolved versus Surface-Associated

Upon binding of cysteine to the Fh surface, its oxidation is coupled to the reduction of structural Fe­(III) in Fh, generating surface-associated Fe­(II) (Fe­(II)_s_). Part of this pool is released as dissolved Fe­(II) (Fe­(II)_aq_; Figure 1), while another fraction undergoes interfacial electron transfer (IET), generating labile surface-associated Fe­(III) (Fe­(III)_s_),^21,22,41^ which acts as a precursor for secondary mineral formation (Figure S2). To evaluate the effect of coprecipitated silica on these processes, we monitored the temporal evolution of Fe­(II)_aq_, Fe­(II)_s_, and Fe­(III)_s_ during the anoxic incubation of Fh, Si-Fh_0.05, and Si-Fh_0.1 in the presence of cysteine (Figure 4).

**Figure 4:**
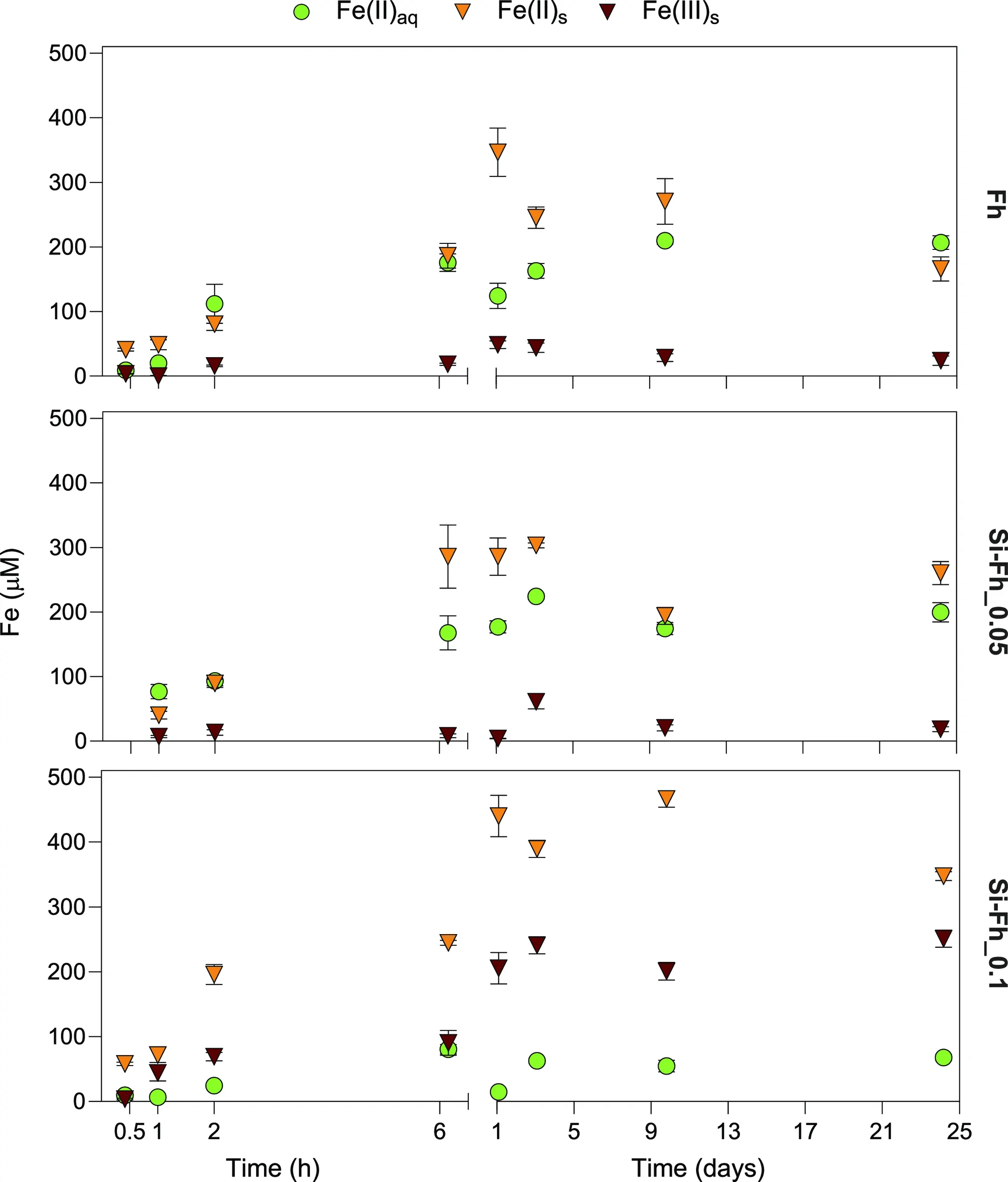
Distribution of Fe species (aqueous-phase Fe­(II)=Fe­(II)_aq_, solid-associated Fe­(II)=Fe­(II)_s_, solid-associated Fe­(III)=Fe­(III)_s_) following incubation of ferrihydrite (top), Si-Fh_0.05 (middle) and Si-Fh_0.1 (bottom) with cysteine under anoxic conditions.

During the first 30 min of incubation, Fe­(II)_aq_ and Fe­(III)_s_ concentrations remained negligible (<10 μM) in both Fh and Si-Fh_0.1, whereas Fe­(II)_s_ concentrations reached ∼40 μM and ∼60 μM, respectively. This indicates that Fe­(II)_s_ forms initially through Fh reduction, and only subsequently partitions into Fe­(II)_aq_ or participates in IET to generate Fe­(III)_s_. The simultaneous increase in Fe­(II)_aq_, Fe­(II)_s_, and Fe­(III)_s_ throughout incubation suggests that these processes proceed concurrently in the presence of all minerals (Figure 4).

Throughout the incubation, Fe­(II)_s_ concentrations remained consistently higher than those of Fe­(II)_aq_ and Fe­(III)_s_, indicating a strong Fe­(II) affinity for the mineral surface (Figure 4). However, distinct differences emerged as a function of the silica loading. Si-Fh_0.05 exhibited Fe species distributions broadly similar to those of Fh, whereas Si-Fh_0.1 showed substantially greater accumulation of surface-associated Fe species, particularly Fe­(III)_s_, which were otherwise negligible. Notably, elevated Fe­(III)_s_ concentrations in Si-Fh_0.1 were already observed at early incubation stages prior to detectable mineral transformation by XRD (Figure 2). This suggests that the increased Fe­(III)_s_ pool cannot be explained solely by differences in affinity among newly formed crystalline phases but instead reflects stabilization of the labile Fe­(III)_s_ pool by higher silica loading.

On the basis of these observations, we propose two silica-dependent mechanisms responsible for delayed or hindered Fh transformation: (1) physical interference with mineral recrystallization by coprecipitated silica and (2) stabilization of the labile surface-associated Fe­(III)_s_ pool, suppressing secondary mineral nucleation and growth (Figure S3). We suggest that at the lower Si/Fe ratio of 0.05, mechanism (1) dominates, resulting in an overall delay and a decrease in crystalline phase generation. In contrast, at the higher Si/Fe ratio of 0.1, both mechanisms operate simultaneously, resulting in the inhibition of crystalline phase formation within the time frame of our experiments. In both cases, reprecipitation and structural reorganization of poorly crystalline Fh likely continue.

Previous studies have shown that silicate inhibits ferrihydrite transformation by binding to FeO_6_ corner-sharing sites, thereby disrupting Fe–O–Fe polymerization during recrystallization and precipitation of more ordered Fe oxyhydroxides.^52^ This mechanism is consistent with the delayed but eventual transformation observed for Si-Fh_0.05. In contrast, the elevated Fe­(III)_s_ concentrations observed for Si-Fh_0.1 suggest an additional stabilization mechanism that suppresses the nucleation of crystalline secondary phases. Similar effects have previously been reported for dissolved organic ligands such as citrate, which stabilize Fe­(III)_s_ through complexation, thereby interfering with mineral nucleation and growth.^17,53,54^ Under such conditions, goethite nucleation may be particularly inhibited because its formation requires sustained availability of Fe­(III)_s_, whereas metastable phases such as lepidocrocite may remain kinetically favored.^17^ Despite suppression of crystalline phase formation in Si-Fh_0.1, cysteine-driven reductive dissolution and IET still proceeded, as evidenced by cysteine oxidation and the accumulation of Fe­(II)_s_, Fe­(III)_s_, and Fe­(II)_aq_ (Figures 1 and 4). These findings indicate that generation of Fe­(II)_s_ alone is insufficient to drive crystallization at elevated silica loading, which appears to decouple reductive electron transfer from subsequent mineral recrystallization pathways.^44^

#### Mass-Balance Calculations

The results above show that cysteine-induced reduction redistributed Fe among dissolved, surface-associated, and newly formed solid-phase pools, yet the Fe-cysteine mass balance did not fully close. Assuming that the mineral surface was coated exclusively by cystine, the near-complete loss of dissolved cysteine would imply complete oxidation. However, stoichiometric predictions compared against measured values reveal a significant mass-balance deficit (∼146–209 μM; Table S5), suggesting that not all adsorbed cysteine was converted to cystine.

We therefore propose that unreacted cysteine coadsorbed with cystine, forming an organic surface layer that may stabilize surface-associated Fe species. Under this framework, the “missing” Fe (∼95–132 μM; Table S5) likely reflects sequestration into phases or pools not captured by the extraction scheme. This Fe may be incorporated into newly formed Fe phases (*e.g.*, acid-resistant lepidocrocite in Fh incubations) or retained within an amorphous Si–Fe–OM matrix^52^ (in Si-Fh incubations).

### Characteristics of Mineral Products

Because coprecipitated silica altered both the extent and pathway of Fh transformation, we further characterized the structure and composition of the resulting mineral products using TEM-EDX imaging, Mössbauer spectroscopy, FTIR, and XPS analysis, at different transformation stages. We focused on the untreated minerals and on transformed Fh and Si-Fh_0.05 products following 6 and 20 days of incubation (Figures 3, 5).

**Figure 5:**
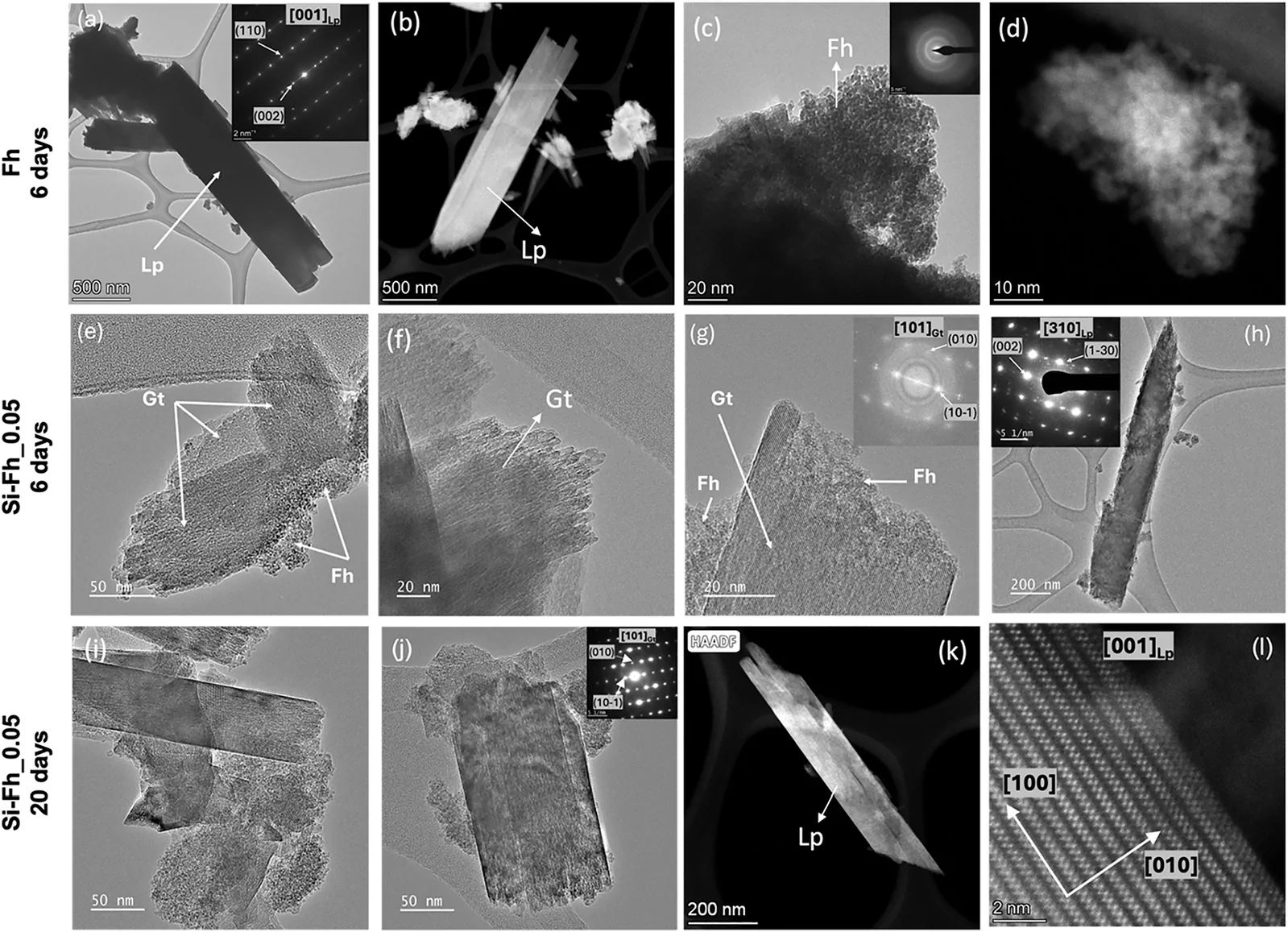
Transmission electron microscopy (TEM) images of mineral products following either a 6- or 20-day incubation of ferrihydrite (Fh; Si/Fe = 0) and Si-Fh_0.05 (Si/Fe = 0.05) with cysteine under anoxic conditions. 6-Day incubation of Fh (top): Panels (a, b) present lepidocrocite (Lp) and panels (c, d) show the residual Fh, where the insets in panels (a) and (c) represent the diffraction rings that confirmed the Lp and Fh phases, respectively. 6-Day incubation of Si-Fh_0.05 (middle): Panels (e–g) show a bifurcated and porous-like goethite (Gt) phase while panel (h) shows an identified Lp particle, with its corresponding diffraction ring in the inset. 20-Day incubation of Si-Fh_0.05 (bottom): Panels (i, j) show the bifurcated, loosely packed Gt, with its corresponding diffraction ring in the inset and panels (k, l) show another large Lp particle.

#### Structure and Crystallinity

Following a 6-day incubation of Fh with cysteine, large, blade-like crystals were observed that corresponded to lepidocrocite, based on selected-area electron diffraction (SAED; Figure 5a,b). The partial transformation was evidenced by residual Fh, present as aggregates of spherical nanoparticles with a poorly crystalline structure (Figure 5c,d), similar to untreated Fh (Figure S8). Earlier work also demonstrated that Fe­(II)-catalyzed transformation of organic-coated Fh results in its partial recrystallization into thin lepidocrocite plates or nanocrystals embedded within an amorphous matrix.^6^

Interestingly, TEM analysis of the 6-day incubated Si-Fh_0.05 revealed significant changes in mineral structure and porosity (Figure 5) that differed from both the 6-day incubated Fh and the untreated Si-Fh_0.05 (Figure S8). Although the sample was expected to consist primarily of poorly crystalline residual Fh nanoaggregates, a considerable quantity of another mineral phase was observed. The particles of this mineral phase consisted of loosely packed but closely oriented crystal domains (Figure 5e–g). They exhibited branched growth fronts and domains of varying lengths. SAED identified this porous-like mineral phase as goethite, in both the 6- and 20-day samples (Figure 5j). Increased particle crystallinity of the extended 20-day incubated Si-Fh_0.05 was evidenced by larger size of crystal domains, well-defined morphology, and surfaces (Figure 5i,j), while the porous-like features persisted.

These observations are consistent with bulk Mössbauer spectroscopy analysis, which detected minor amounts of goethite (Figure 3 and Table S4) in the 6-day incubated Si-Fh_0.05. In addition, several lepidocrocite particles were observed via TEM of the 6-day Si-Fh_0.05 sample (Figure 5h). While these were below the detection limit of XRD, their presence aligns with the Mössbauer results, confirming a minor contribution of this phase to the mineral mixture.

#### Spatial Distribution of Elements: C, S, and Si

Organic coatings on Fh and Si-Fh_0.05 surfaces were evidenced by elevated C, S, and N levels determined by XPS (Figure S10), together with the appearance of characteristic organic functional groups by FTIR (Table S6 and Figure S11), relative to the untreated minerals. After a 6-day anoxic incubation of Fh with cysteine, elemental mapping unveiled a distinctly higher S content in residual Fh (S/Fe = 0.087) compared to the newly formed lepidocrocite (negligible S content; Figure 6a-a3). The elevated S content is attributed to cysteine- and/or cystine-derived organic coatings preferentially associated with residual Fh rather than newly formed lepidocrocite (Figures 6a3 and S9). This preferential association likely reflects the higher surface area and porosity of Fh relative to lepidocrocite.^55^ Ultimately, lepidocrocite showed minor organic association (Figures 6a1 and S9) regardless of the precursor Fh mineral (i.e., Fh or Si-Fh) and incubation time, contributing to the occurrence of “patchy” organic-rich Fh versus organic-poor lepidocrocite domains (Figure 6a1).

**Figure 6:**
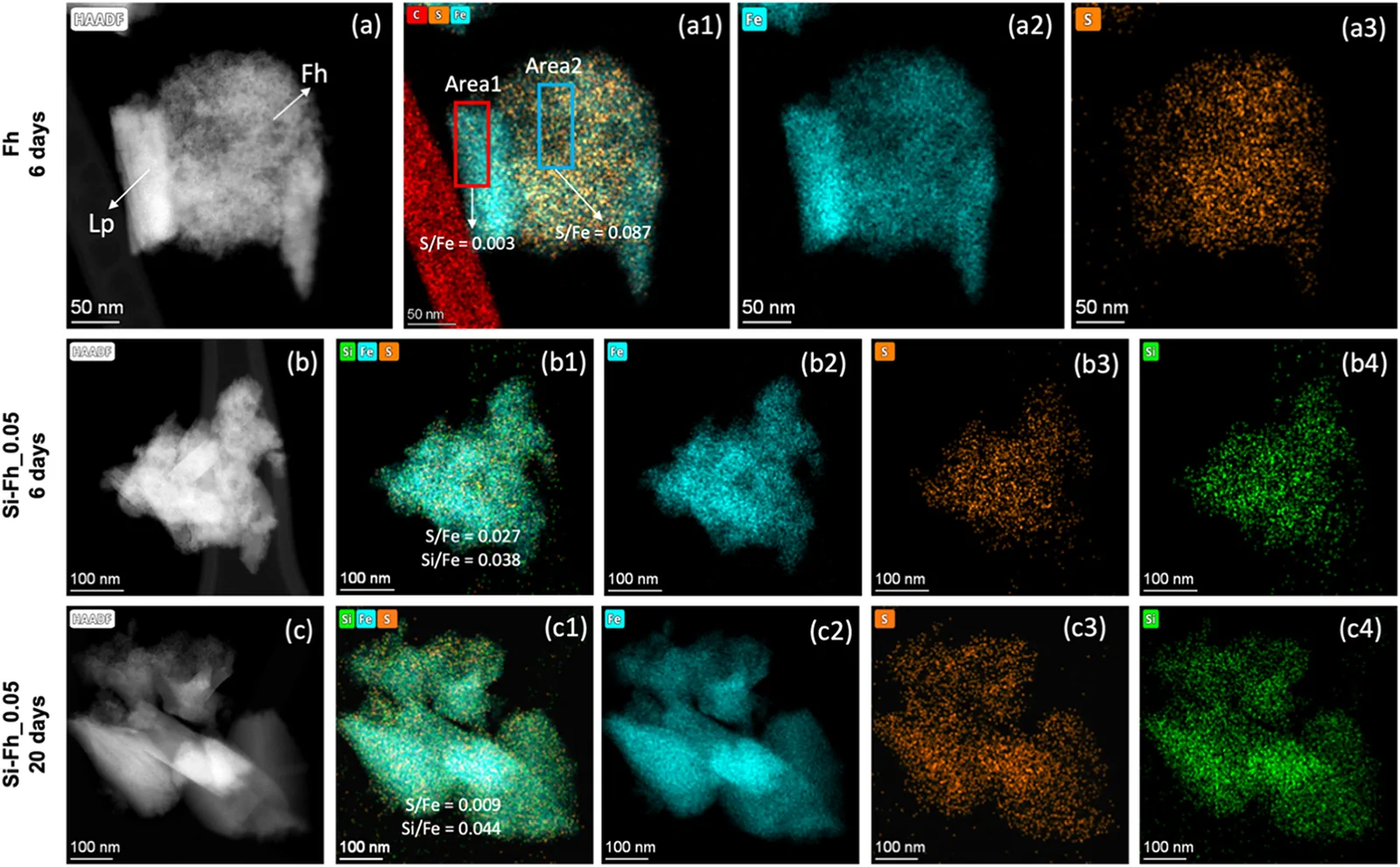
Transmission electron microscopy (TEM) images and corresponding energy-dispersive X-ray (EDX) elemental maps of mineral products following either a 6- or 20-day incubation of ferrihydrite (Fh) and Si-Fh_0.05 with cysteine under anoxic conditions. 6-Day incubation of Fh (top): Panel (a) presents Fh partially transformed into Lp with corresponding EDX maps of Fe, S, and C in panels (a1–3), where the red section on the left of panel (a1) is attributed to C that originates from the TEM grid. 6-Day incubation of Si-Fh_0.05 (middle): Panel (b) presents porous-like goethite (Gt) with corresponding EDX maps of Fe, S, and Si in panels (b1–b4). 20-Day incubation of Si-Fh_0.05 (bottom): Panel (c) presents porous-like Gt with corresponding EDX maps of Fe, S, and Si in panels (c1–c4).

Unlike lepidocrocite, the porous-like goethite phase that evolved from the anoxic incubation of Si-Fh_0.05 with cysteine displayed higher S contents, which appeared to decrease upon mineral aging (e.g., from S/Fe of 0.027 after 6 days to 0.009 after 20 days; Figure 6). This suggests that progressive goethite crystallization is accompanied by redistribution and partial release of adsorbed organics, likely due to loss of reactive surface area and sorption sites during transformation of high-surface-area Fh into more crystalline goethite (Figure S10).

Silica distribution in untreated Si-Fh_0.05 was spatially homogeneous, with an average Si/Fe ratio of 0.05 ± 0.01 (Table S1 and Figure S8). Similarly, Si remained uniformly distributed within the porous-like goethite phase after both 6 and 20 days of incubation, with Si/Fe ratios of 0.038 and 0.044, respectively, remaining close to the initial value of 0.05 (Figure 6b1,c1). These observations indicate retention of silica within the porous goethite phase during Fh transformation. Similar retention has been reported for high affinity oxyanions during Fh transformation, where phosphate inhibited reductive recrystallization and influenced secondary Fe mineral formation during quinone-driven Fh transformation.^56^

Contrary to the loosely packed goethite phase, lepidocrocite generated from Si-Fh_0.05 exhibited lower silica association, with Si/Fe ratios decreasing to ∼0.018 after 20 days (Figure S9). These contrasting associations of silica and organic compounds with lepidocrocite versus goethite are another indication of the differences in Fe mineral formation pathways resulting from the silica association. In the absence of silica, the continuous Fe­(II)/Fe­(III)-dissolution–precipitation promotes the growth of large blade-like lepidocrocite crystals that contain little Si or S.^11,57^ In contrast, the disruptive silica associations in Si-Fh_0.05 may direct slower recrystallization of nanoscale goethite domains with different growth orientations that do not undergo Ostwald ripening.^58,59^

## Environmental Implications

Our findings demonstrate that coprecipitated silica acts as a critical regulator of Fh transformation dynamics under anoxic environments. Specifically, silica loading dictates the rate and extent of Fh transformation as well as the identity and characteristics of the secondary mineral products. Mechanistically, lower Si/Fe ratios primarily inhibit recrystallization through physical interference, whereas higher Si/Fe ratios also stabilize labile surface-associated Fe­(III) to suppress the crystalline phase formation. These mechanisms may extend to other metastable Fe phases undergoing aging processes such as mackinawite and green rust. By preserving amorphous structures and directing recrystallization toward highly porous phases, coprecipitated silica helps to maintain the extensive surface areas required for the preservation of MAOM. Therefore, prolonged incubations of months or years, accompanied by comprehensive temporal evaluation of mineral products, are required to fully understand the role silica plays in Fe mineral aging and the potential for MAOM persistence.

## Supplementary Material



## Notes

Table S1 presents Si/Fe molar ratios of Si-Fh minerals; Table S2 presents concentrations of cysteine and cystine in Fh-free control samples; Table S3 presents semiquantitative mineral phase distributions derived from Rietveld refinement of XRD data; Table S4 presents Mössbauer parameters; Table S5 presents mass-balance data, and Table S6 presents FTIR peak assignments. Figures S1–S11 include Rietveld refinement validation, a cysteine-Fh reaction scheme, a conceptual scheme explaining the effect of silica loading on Fh transformation, aqueous-phase Si levels and pH values, Mössbauer spectra for untreated minerals and transformation products at select time points, cystine adsorption isotherms by Fh and lepidocrocite, TEM and TEM-EDX images of untreated minerals and a lepidocrocite product, XPS analysis and FTIR spectra of untreated and incubated minerals (PDF)
