# Supplementary material for: Coprecipitated Silica Controls the Divergent Pathways of Cysteine-Induced Ferrihydrite Transformation
Source: Environ Sci Technol. 2026 Aug 7;60(32):22466–76. doi: 10.1021/acs.est.6c02208 (PMC13492282; doi:10.1021/acs.est.6c02208)
Supplement: Supplementary file 1 [file es6c02208_si_001.pdf]

Supporting Information for

Coprecipitated silica controls the divergent pathways of cysteine-induced ferrihydrite transformation

Yinyin Zheng [a], Libor Kovarik [b], Ravi K. Kukkadapu [b], Qian Zhao [b,c] and Maya Engel [a,*]

[a] Department of Soil and Water Sciences, The Robert H. Smith Faculty of Agriculture, Food and Environment, The Hebrew University of Jerusalem, Rehovot, 7610001, Israel

[b] Environmental Molecular Sciences Laboratory, Pacific Northwest National Laboratory, Richland, WA, 99354, United States

[c] Department of Sustainable Earth Systems Sciences, The University of Texas at Dallas, Richardson, TX, 75080, United States

Table of Contents

Details on methodology and analysis ···· **3**

Section S1. High-performance liquid chromatography (HPLC) ···· 3

Section S2. X-ray diffraction (XRD) ···· 4

Section S3. Transmission electron microscopy (TEM) ···· 5

Section S4. X-ray photoelectron spectroscopy (XPS) ···· 6

Section S5. Fourier-Transform infrared (FTIR) spectroscopy ···· 7

Section S6. Mössbauer spectroscopy ···· 8

Section S7. Cystine adsorption isotherms ···· 10

Table S1. Si/Fe molar ratios of Si-Fh minerals ···· **11**

Table S2. Concentrations of cysteine levels in solution ···· **12**

Table S3. Quantitative mineral phase distribution from XRD ···· **13**

Table S4. Calculated Mössbauer spectral parameters of untreated and incubated minerals ···· **14**

Table S5. Mass balance calculations of Fe and organic species ···· **16**

Table S6. FTIR peak assignments for untreated and incubated minerals ···· **17**

Figure S1. Rietveld refinement validation using known mineral mixtures ···· **18**

Figure S2. Schematic representation of cysteine-induced Fh transformation ···· **19**

Figure S3. Proposed mechanism for silica regulated Fh transformation ···· **20**

Figure S4. pH and dissolved Si concentrations during Fh and Si-Fh incubations ···· **21**

Figure S5. Mössbauer spectra (12 K, 60 K, 77 K, and 80 K) of untreated minerals ···· **22**

Figure S6. Low-temperature Mössbauer spectra (12 K) of untreated and incubated minerals ···· **23**

Figure S7. Cystine adsorption isotherms by Fh and Lp fitted by the Langmuir model ···· **24**

Figure S8. TEM-EDX images and diffractions rings of untreated Fh and Si-Fh ···· **25**

Figure S9. TEM-EDX images of Lp after 20 days of incubation of Si-Fh_0.05 ···· **26**

Figure S10. XPS analysis of untreated and incubated minerals ···· **27**

Figure S11. FTIR spectra of untreated and incubated minerals, cysteine and cystine ···· **28**

Bibliography ···· **29**

Details on methodology and analysis

Section S1. High-performance liquid chromatography (HPLC)

Cysteine and cystine concentrations were determined after derivatization with fluorenylmethyloxycarbonyl chloride (FMOC-Cl) using a Vanquish HPLC system (Thermo Fisher Scientific, USA) equipped with a Kinetex EVO C18 column (2.6 µm, 100 Å, 100 × 4.6 mm; Phenomenex). Derivatization was performed by mixing 100 µL of each sample dilution with 500 µL of 0.5 M borate buffer (pH 7.4) and 400 µL of 200 mg/L FMOC-Cl solution, followed by shaking at room temperature for 30 min. Subsequently, 100 µL of the derivatized mixture was diluted with 900 µL of 30% acetonitrile and 70% double-deionized water (DDW) containing 0.1% formic acid. Separation was carried out at 40 °C using a binary mobile phase consisting of buffer A (DDW with 0.1% formic acid) and buffer B (acetonitrile) at a flow rate of 0.4 mL/min. The elution program was as follows: 30% B from 0–3.0 min, increased linearly to 88% B at 15.5 min, then to 100% B at 17.5 min, returned to 30% B at 18.0 min, and held until 28.0 min for re-equilibration. Cysteine- and cystine-FMOC derivatives were detected at 265 nm at 12.4 min and 13.5 min, respectively. Limits of detection (LOD) and quantification (LOQ), based on signal-to-noise ratios of 3 and 7, were 0.017 mg/L (0.140 µM) and 0.039 mg/L (0.326 µM) for cysteine, and 0.085 mg/L (0.356 µM) and 0.199 mg/L (0.831 µM) for cystine, respectively.

Section S2. X-ray diffraction (XRD)

Rietveld refinement. Rietveld refinement was employed for quantitative phase analysis and structural interpretation of the polycrystalline materials[1–3]. Crystalline phases were identified and quantified using TOPAS software, including lepidocrocite (PDF 00-044-1415) and goethite (PDF 01-073-6522) minerals. The total degree of crystallinity was subsequently used to estimate the fraction of amorphous material (*i.e.*, Fh or Si-Fh), from which the relative abundances of lepidocrocite (Lp) and/or goethite (Gt) were determined. The fit quality was judged based on the goodness of fit (GOF), calculated as R_{wp}/R_{exp} in TOPAS, with values <2 indicating excellent fit quality[4].

Validation. This data processing pipeline was validated by applying it to mixtures of known mass ratios of Fh, Lp, and Gt (including Fh:Lp:Gt proportions of 95:5:0, 90:10:0, 80:10:10, 60:20:20, 40:30:30, and 20:40:40). The results showed that the fitting was satisfactory to identify the amorphous (*i.e.*, Fh or Si-Fh) versus crystalline content, however where both Lp and Gt phases co-existed, the uncertainty in their contribution increased (Fig. S1). It appeared that the amorphous Fh phase was slightly underestimated at contributions >70% and overestimated at its lower contributions. Given these limitations and uncertainties of the Rietveld refinement, we use the fitting results as semi-quantitative rather than exact. Nonetheless, because the differences between Fh and Si-Fh minerals are evident in the diffractograms, we propose that together with the TEM and Mössbauer findings, these phase calculations may be used to understand the trends over time and evaluate the impact of the coprecipitated silica on mineral transformation dynamics.

Section S3. Transmission electron microscopy (TEM)

Structural observations were performed with Thermo Fisher Scientific microscopes, including probe corrected Themis Z 30–300, probe corrected Spectra 30–300 and image corrected Environmental Titan 80–300. All observations were performed at 300kV. The environmental Titan 80–300 was primarily used for high resolution phase contrast imaging and diffraction measurements under low dose conditions. The observations were performed with a Gatan Metro 300 state-of-the-art single electron counting camera. Themis Z 30–300 and Spectra were primarily used for EDX analysis, which are equipped with a Super-X G2 EDX detector, and an Ultra-X Spectra EDX detector, respectively. EDX acquisitions were made with probe currents of approximately of 50 pA for the different samples analyzed. All maps were obtained with multi-frame drift corrected acquisition. EDX data processing was performed with Velox software from Thermo Fisher Scientific.

Analysis of acquired images and diffraction patterns was performed using Gatan Digital Micrograph. Crystallographic analysis was done using Crystal Maker and Single Crystal software from CrystalMaker Software Ltd. We note that the indexing of geothite electron diffraction patterns was performed in the context of space group Pnma (No. 62). Alternatively, the structure is sometimes referred in the literature under a different cell choice, defined as Pbnm (No. 62).

Section S4. X-ray photoelectron spectroscopy (XPS)

Sample analysis. Elemental composition on the mineral surface was analyzed using X-ray photoelectron spectroscopy (XPS) at the Environmental Molecular Sciences Laboratory (EMSL) in Washington, USA by mounting the dry mineral powders onto Si wafers. Spectra were collected on a Physical Electronics Quantera Scanning X-ray Microprobe (Physical Electronics, Germany) equipped with a monochromatic Al Kα (1486.7 eV) X-ray source. X-rays were directed perpendicular to the sample surface, and photoelectrons were collected at a 45° detection angle. Binding energies were determined with an accuracy of ±0.2 eV, referenced to the C 1s peak at 284.8 eV. High-resolution spectra for quantitative analysis were acquired using a pass energy of 69 eV with a step size of 0.125 eV.

Interpretation of results. The XPS analysis of Si-Fh samples showed a surface Si/Fe ratio of 0.09 (Table S1 and Fig S10). Although a minor signal was detected in the untreated Fh (Si/Fe=0.04), the detection of Si in the Fh sample is likely an artifact resulting from the use of a silicon wafer as the sample holder. Since no Si was intentionally added during the synthesis of Fh, this background Si signal should not be interpreted as genuine surface-associated Si.

Section S5. Fourier-Transform infrared (FTIR) spectroscopy

Sample analysis. Chemical bonding and functional group composition were assessed using an FTIR spectrophotometer (Nicolet 6700, Thermo), equipped with an attenuated total reflectance (ATR) accessory. Spectra were collected over the range of 4000–400 cm^{-1} with 32 scans per sample.

Interpretation of results. FTIR spectra of the untreated Fh and Si-Fh_0.1 minerals were dominated by broad bands at ~3000–3500 cm^{-1} and ~1620 cm^{-1}, which likely correspond to O–H stretching vibrations and H–O–H bending, respectively (Table S6 and Fig. S11). The untreated Si-Fh_0.1 sample additionally exhibited a distinct band near ~930 cm^{-1}, attributed to Si–O–Fe vibrations, consistent with the association of silica with Fh[5,6] . A weak band near ~1480 cm^{-1} was also observed in the untreated Si-Fh_0.1 sample and may be assigned to surface hydroxyl bending vibrations.

Following a 3-day incubation with cysteine under anoxic conditions, additional bands associated with organic interactions appeared in both Fh and Si-Fh_0.1 samples (Fig. S11). These included aliphatic C–H stretching vibrations (~2840–2900 cm^{-1}) and COO^- symmetric stretching bands (~1380–1390 cm^{-1}), indicating association of cystine/cysteine with the mineral surfaces. The incubated Fh sample further exhibited bands near ~1020 and ~1160 cm^{-1}, assigned to C–N and/or C–O stretching vibrations, as well as a band near ~746 cm^{-1} tentatively attributed to C–S and/or NH_2 wagging vibrations. In the incubated Si-Fh_0.1 sample, the ~930 cm^{-1} Si-associated band remained present, suggesting preservation of Si-related structural features following incubation.

Section S6. Mössbauer spectroscopy

Sample selection. Incubated mineral products were selected for Mössbauer spectroscopy analysis based on the following rationalization:

- We had limited time for Mössbauer analysis as this resource was used as part of an EMSL Exploratory research grant.
- Based on the XRD results showing negligible secondary mineral formation upon incubation of the Si-Fh_0.1 mineral (Fig. 2), we decided to utilize Mössbauer spectroscopy to analyze and compare the Fh and Si-Fh_0.05 incubated minerals.
- We first collected spectra of the untreated Fh and Si-Fh_0.05 minerals.
- After obtaining TEM results of the 6-day incubated Si-Fh_0.05 mineral (Fig. 5) showing the porous-like and bifurcated goethite phase, which was not identified through XRD, we decided to analyze the 6-day incubated Fh and Si-Fh_0.05 minerals.
- Lastly, based on the combined XRD, Mössbauer, and TEM results of the Si-Fh_0.05 mineral, we decided to use the remaining Mössbauer spectroscopy allocated time to characterize the incubated products at a short (1 h) and long (20 days) timepoint. This provided us with insights into the mineral phase distribution dynamics over time.

Interpretation of results. We chose to analyze the Mössbauer spectra at 60 K because at 77 K, both ferrihydrite (Fh) and lepidocrocite display similar doublet features and are not easily distinguished. Furthermore, at 12 K, we cannot unambiguously differentiate the relative contributions of Fh/Si-Fh and goethite (Fig. S5), particularly if the contribution of one of these phases is minor, for example, less than 10%. Therefore, in this study, we use 60 K Mössbauer results to quantify phase changes upon cysteine-induced Fh transformation (Fig. 3).

To track bulk changes in Fh crystallinity at 60 K, the Fh phase is modeled into *component 1* and *component 2* (Table S4), even though these bulk differences are not readily apparent in the TEM images (Fig. 5). As in the 60 K spectrum (Fig. 3), the two Fh *components* and lepidocrocite peaks are evident in the corresponding 12 K spectrum (Fig. S6). It is plausible that a small fraction of Fe in the *components* of Fh is Fe(II), which is undetectable by Mössbauer spectroscopy, as suggested by Zachara et al. (2007)[7]. The mixture of relatively higher crystalline Fh, lepidocrocite, and goethite crystals, as well as the presence of organic coatings on Fh surfaces but not on lepidocrocite and goethite crystals, demonstrates the cysteine-induced catalytic reductive transformation of the initial Fh.

Section S7. Cystine adsorption isotherms

Adsorption experiments. Batch adsorption experiments were conducted to determine the adsorption affinity and capacity of cystine on Fh and lepidocrocite (Lp). The Lp was synthesized based on the procedures outlined by Schwertmann and Cornell (2008)[8], and the phase purity of the resulting Lp formation was validated using XRD (results not shown). For each sample, 25 mg of the respective mineral was suspended in 20 mL of solution. A series of initial cystine concentrations was used, ranging from 10 mg/L to 110 mg/L. The suspensions were equilibrated for 24 hours to ensure complete interaction. The solid and liquid phases were separated using a 0.45 μm PES syringe filter. The concentration of sulfur in the filtrate was measured by inductively coupled plasma optical emission spectroscopy (ICP-OES) to determine the equilibrium concentration of cystine (C_e). The amount of cystine adsorbed per unit mass (q_e) was calculated from the mass balance between the initial and equilibrium concentrations.

Langmuir isotherm fitting. The adsorption data were analyzed using the Langmuir model, which assumes monolayer adsorption on a homogeneous surface with a finite number of identical sites. The non-linear form of the Langmuir equation was fitted to the experimental data using the following equation (Eq. 1):

$$q_e = \frac{q_{max} K_L \mathrm{C_e}}{1 + K_L \mathrm{C_e}} \quad (1)$$

Where q_e is the amount of cystine adsorbed at equilibrium (μmol/g), C_e is the equilibrium concentration of cystine (μmol/L), q_{max} represents the maximum adsorption capacity of the mineral (μmol/g), and K_L is the Langmuir affinity constant (L/μmol). Non-linear regression analysis and curve fitting were performed using GraphPad Prism.

Table S1. *Si/Fe molar ratios in ferrihydrite (Fh) and in the Si-Fh minerals determined using TEM-EDX, SEM-EDX, ICP-OES, and XPS. Values represent average measured Si/Fe ratios (conducted in triplicates) from each method.*

**Mineral**	**TEM-EDX**	**SEM-EDX**	**ICP-OES**[1]	**XPS**[2]	***Average***[3]
Fh	0	0	0	0.04	*negligible*
Si-Fh (low Si/Fe)	0.05	0.05	0.04	0.09	*0.05* ± *0.01*
Si-Fh (high Si/Fe)	0.11	0.09	0.10	0.18	*0.10* ± *0.01*

[1] For ICP-OES analysis, Si-Fh coprecipitates were acid-digested (concentrated HNO_3 (65%), HCl (30%), and H_2O_2 (30%)) prior to Fe and Si quantification;[2] Si/Fe ratios obtained by XPS were not included in the average calculation because XPS reflects surface elemental composition rather than bulk mineral composition; [3] Average values are reported as mean ± standard deviation calculated from the three analytical methods (TEM-EDX, SEM-EDX, ICP-OES).

Table S2. *Concentrations of cysteine and cystine in mineral-free control samples (run in parallel to the samples in the presence of ferrihydrite (Fh), Si-Fh_0.05, and Si-Fh_0.1 minerals).*

Mineral	Time (days)	Cysteine (μM)	Cystine (μM)
**Fh**	0	1131.8±5.7	17.6±15.2
	0.2	1299.5±109.3	0±3.0
	1	1138.5±24.0	0.2±0.0
	6	1124.5±15.4	0.0±0.0
**Si-Fh_0.05**	0	1211.4±28.9	2.1±0.1
	0.2	1261.6±22.9	6.5±1.8
	1	1190.5±18.0	21.9±0.3
	7	1133.2±28.5	28.6±0.8
	10	910.1±148.2	46.0±11.2
**Si-Fh_0.1**	0	1119.0±26.6	13.2±11.1
	0.2	1099.3±48.7	64.1±0.3
	1	910.2±111.8	56.5±16.4

Table S3. *Fitting results of the Rietveld refinement of XRD patterns following incubation of ferrihydrite (Fh), Si-Fh_0.05, and Si-Fh_0.1 minerals with cysteine under anoxic conditions.*

Mineral	Time (days)	Mineral phase distribution (%)			GOF [a]
		Ferrihydrite	Lepidocrocite	Goethite	
**Fh**	0	100	0	0	-
	3	88	12	0	1.09
	6	81	19	0	1.36
	10	68	32	0	1.26
	20	62	38	0	1.17
**Si-Fh_0.05**	0	100	0	0	-
	3	100	0	0	-
	6	100	0	0	-
	10	76	7	17	1.06
	20	71	8	21	1.08
**Si-Fh_0.1**	0.2	100	0	0	-
	1	100	0	0	-
	3	100	0	0	-
	10	100	0	0	-
	17	100	0	0	-

[a] Goodness of fit (GOF) is defined as R_{wp}/R_{exp}, where R_{wp} is the weighted profile R-factor and R_{exp} is the expected R-factor.

Table S4. *Fitted Mössbauer spectral parameters (at 60 K and 12 K) of 2-line ferrihydrite (Fh) and Si-Fh_0.05 following incubation with cysteine under anoxic conditions.*

**Mineral**	**Temp.**	**Phase**[1]	**<CS>**[2] *mm/s*	**<ε>**[3] *mm/s*	**<|H|>**[4] *Tesla*	**sHFD**[5] *Tesla*	**Component** [6]%	**phase**[7] %	χ^2[8]
Untreated Fh	60	Fh	0.48	-0.01	24.6	11.03		100	1.5
		Component 1			*30.6*	*8.6*	*62.5*		
		Component 2			*14.4*	*6.5*	*37.5*		
	12	Fh	0.48	-0.01	47.9	2.9		100	3.5
		Component 1			*49.6*	*1.6*	*46.6*		
		Component 2			*46.5*	*2.9*	*53.4*		
Fh; 6-day anoxic incubation with cysteine	60	Fh	0.5	-0.006	27.4	12.1		82.2	1.3
		Component 1			*37.8*	*3.9*	*21.6*		
		Component 2			*24.3*	*12.6*	*78.4*		
		Goethite	0.5	-0.09	49.7	0.3		3.9	
		Lepidocrocite	0.51	0.02	42.2	1		13.9	
	12	Fh	0.48	-0.02	47.5	3.1		43	2.7
		Component 1			*50.2*	*1.1*	*33.4*	41	
		Component 2			*46.2*	*2.9*	*66.6*		
		Lepidocrocite	0.5	0.02	45.3	0.001		15.5	
Untreated Si-Fh_0.05	60	Si-Fh	0.48	-0.003	23.7	11.3		100	1.7
		Component 1			*30.6*	*8.7*	*58.2*		
		Component 2			*14*	*6.8*	*41.8*		
	12	Si-Fh	0.49	-0.006	47.8	2.8		100	3.5
		Component 1			*49.5*	*1.6*	*47.7*		
		Component 2			*46.5*	*2.7*	*52*		
Si-Fh_0.05; 1-hr anoxic incubation with cysteine	60	Si-Fh	0.47	-0.02	27.6	11.6		100	1.6
		Component 1			*35.3*	*7.5*	*57*		
		Component 2			*17.3*	*7.6*	*43*		
	12	Si-Fh	0.49	-0.005	48	2.8		100	3
		Component 1			*49.2*	*1.8*	*61*		
		Component 2			*46*	*2.7*	*38*		

Table S4 continued.

**Mineral**	**Temp.**	**Phase**[1]	**<CS>**[2]	**<ε>**[3]	**<|H|>**[4]	sHFD[5]	**Component %**[6]	**phase %**[7]	$\chi^{2,8}$
Si-Fh_0.05; 6-day anoxic incubation with cysteine	60	Si-Fh	0.49	0.001	22.4	12.0		90.7	1.1
		Component 1			*34.4*	*7.5*	*35*		
		Component 2			*15.7*	*9*	*65*		
		Goethite	0.47	-0.11	49.6	1.2		6	
		Lepidocrocite	0.51	0.02	42.2	2		3.3	
	12	Si-Fh	0.48	-0.02	48	2.6		94.3	1.5
		Component 1			*49.2*	*1.6*	*63.7*		
		Component 2			*45.8*	*2.5*	*36*		
		Goethite	0.56	-0.06	50.8	0.006		5.6	
Si-Fh_0.05; 20-day anoxic incubation with cysteine	60	Si-Fh	0.41	-0.1	19.4	13.8		88	1.6
		Component 1			*40.7*	*7*	*8*		
		Component 2			*13.5*	*17*	*92.1*		
		Goethite	0.5	-0.11	49.5	0.83		12	
	12	Si-Fh	0.49	-0.03	47.7	2.7		85.1	1
		Component 1			*49.4*	*1.4*	*49.9*		
		Component 2			*46*	*2.7*	*50.1*		
		Goethite	0.56	-0.01	50.3	0.6		14.9	

[1] Mineral phase; [2] Center shift relative to α-Fe; [3] Quadrupole shift parameter; [4] Absolute average HF; [5] Standard deviation of hyperfine field distribution (HFD); [6] Relative distribution of ferrihydrite and Si-ferrihydrite components [7] Relative distribution of various phases; [8] Goodness of fit.

Lorentzian half-width at half maximum (HWHM) of all the elemental sextets is fixed at 0.2 mm/s. The A+/A- areas of the doublet are fixed at 1; coupling between Δ and HF is not allowed, and A1/A3 and A2/A3 are fixed at 3 and 2, respectively; Modeling: Voigt-based structural fitting routine [9].

Table S5. *Mass balance calculations of organic and Fe species following 25 days of anoxic incubation of cysteine with ferrihydrite (Fh) and Si-Fh_0.1. Two conceptual scenarios were tested: (1) complete cysteine oxidation with exclusive cystine adsorption, and (2) partial cysteine oxidation with mixed cysteine and cystine adsorption, based on the reaction:* $2Cysteine + 2Fe^{3+}_{(in\ Fh)} \rightarrow 2Fe^{2+}_{(surface\ associated\ or\ dissolved)} + 2H^{+} + Cystine$

Organic and Fe species	Fh	Si-Fh_0.1	Calculations
Initial cysteine (μM)	1131.8	1119.0	
Total cysteine disappearance (μM)	1119.2	1117.7	Initial cysteine – dissolved cysteine
Total accountable cystine[1] (μM)	350.4	412.1	dissolved cystine + adsorbed cystine
Total accountable Fe[2] (μM)	568.5	728.7	Fe = $Fe(II)_{aq}$ + $Fe(II)_s$ + $Fe(III)_s$
**Scenario 1: Complete cysteine oxidation** *Assumption: All adsorbed cysteine was oxidized to cystine*			
Calculated cystine production (μM)	559.6	558.9	0.5 x cysteine disappeared
"Missing" cystine (μM)	209.2	146.8	calculated cystine – accountable cystine
**Scenario 2: Partial cysteine oxidation** *Assumption: Co-occurrence of adsorbed cysteine and cystine*			
Calculated adsorbed cysteine (μM)	418.3	293.6	2 x "missing" cystine
Calculated Fe production (μM)	700.9	824.1	disappeared cysteine – adsorbed cysteine
"Missing" Fe (μM)	132.4	95.4	calculated Fe – accountable Fe

[1] Calculated as the sum of dissolved (=aqueous) cystine and adsorbed cystine (based on the maximum adsorption capacity obtained from Langmuir isotherm fittings; Fig. S7).

[2] Calculated as the sum of reactive Fe, measured as the sum of $Fe(II)_{aq}$, $Fe(II)_s$, and $Fe(III)_s$ (Fig. 4).

Table S6. *Summary of peaks detected by FTIR analysis (presented in Fig. S11) of untreated ferrihydrite (Fh) and Si-Fh_0.1, and their respective samples following a 3-day incubation with cysteine under anoxic conditions.*

**Functional group / Chemical bond**[5,10]	**Range (cm^{-1})**	**Mineral**			
		**Untreated Fh**	**Untreated Si-Fh_0.1**	**Fh: 3-day incubation**	**Si-Fh_0.1: 3-day incubation**
O–H / N–H / Si–O–H stretch	~3000 – 3500	✓ (~3000)	✓ (~3200)	✓ (~3000)	✓ (~3200)
C–H stretch	~2800 – 2990	—	—	✓ (~2840)	✓ (~2900)
H–O–H bend / COO^- asymmetric stretch	~1580 – 1630	✓ (~1620)	✓ (~1620)	✓ (~1580)	✓ (~1620)
Surface O–H bend	~1480	—	✓ (~1480)	—	—
COO^- symmetrical stretch	~1380 – 1390	—	—	✓ (~1390)	✓ (~1380)
C–N / C–O stretch	~1020 – 1160	—	—	✓ (~1020, 1160)	—
Si–O–Fe stretch	~900 – 1000	—	✓ (~930)	—	✓ (~930)
C–S stretch / NH_2 wagging	~746	—	—	✓ (~746)	—

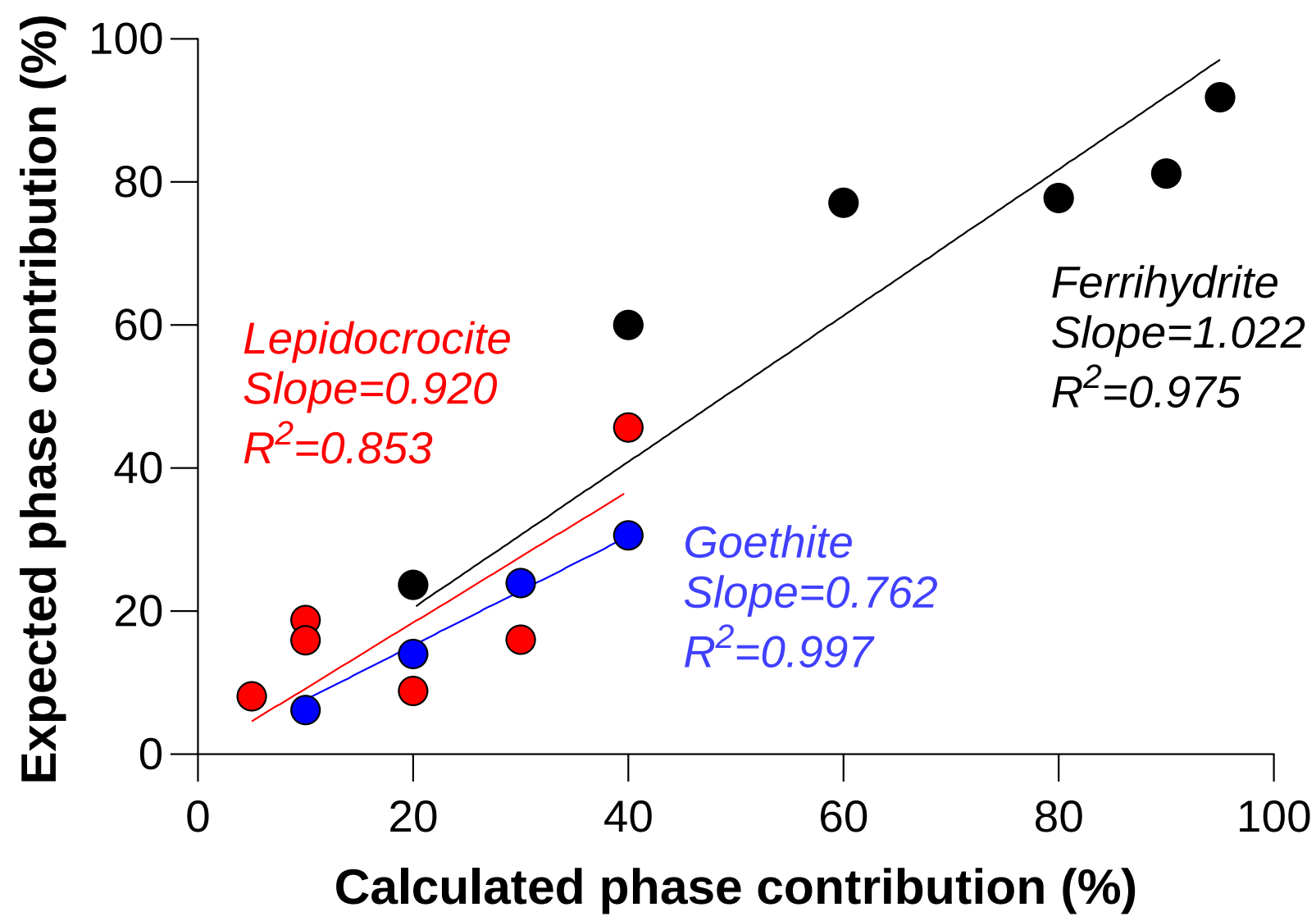

Figure S1. *Mineral phase distribution of known mass ratios of ferrihydrite, lepidocrocite, and goethite mixtures compared to the calculated contributions derived from Rietveld refinement. The slope of the linear regression illustrates how accurate the calculations are, and whether overestimations or underestimations are expected.*

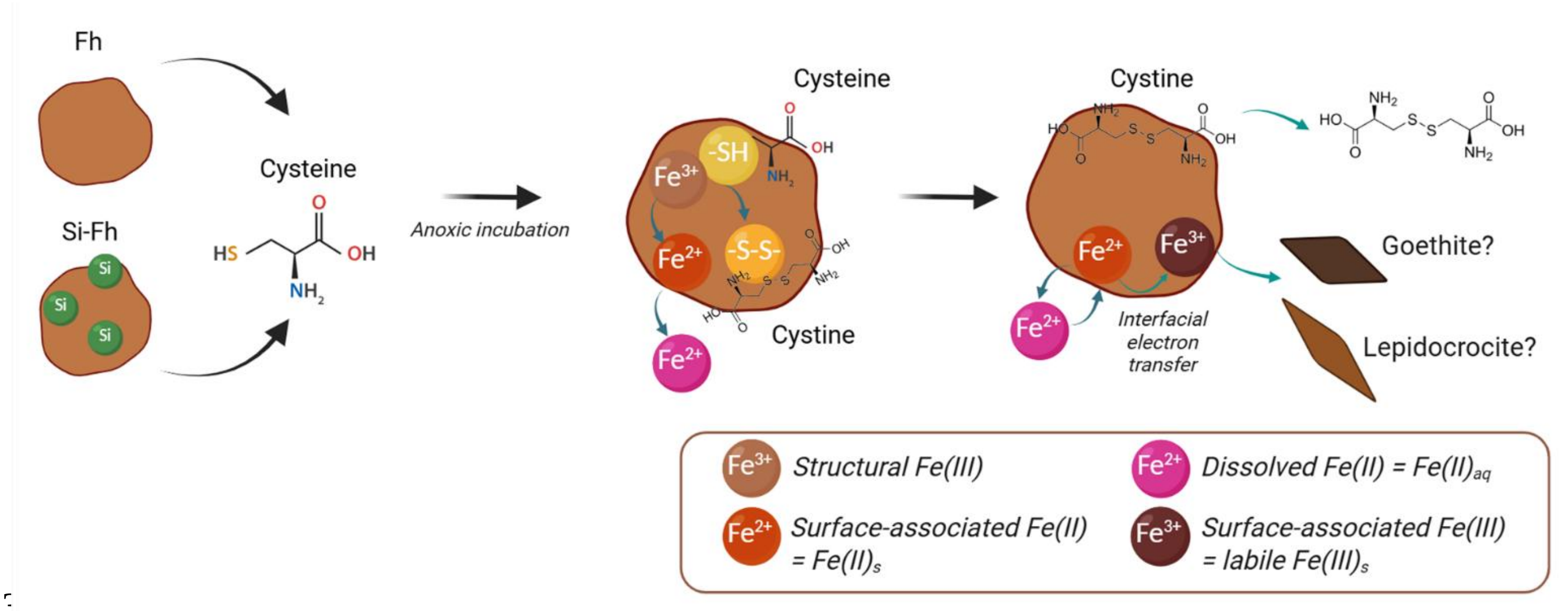

Figure S2. *Schematic representation of the reaction of ferrihydrite (Fh) or Si-Fh with cysteine under anoxic conditions, resulting in Fe(II)-catalyzed Fh mineral transformation. Brown irregular particles represent ferrihydrite aggregates, and silica is shown schematically as surface-associated rather than structurally incorporated.*

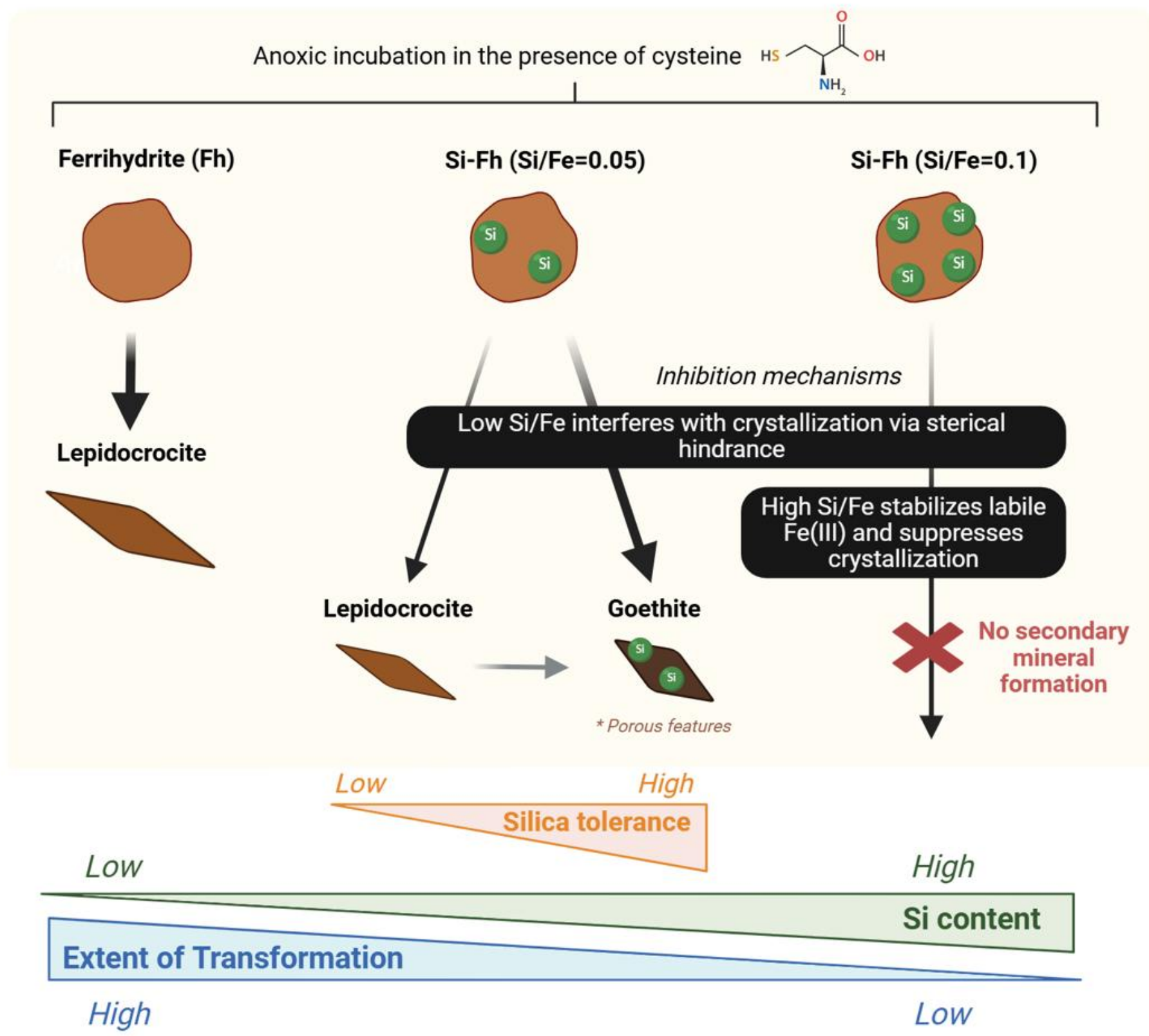

241

242 ***Figure S3.*** *Conceptual scheme of cysteine-induced ferrihydrite (Fh) transformation under anoxic conditions as affected by coprecipitated silica. Increasing Si loading suppresses the extent of transformation, shifting the dominant products from lepidocrocite in Si-free Fh to lepidocrocite/goethite at Si/Fe=0.05, while strongly limiting secondary mineral formation at Si/Fe=0.1. The scheme summarizes the proposed relationship between Si content, extent of Fh transformation, and mineral product formation.*

248

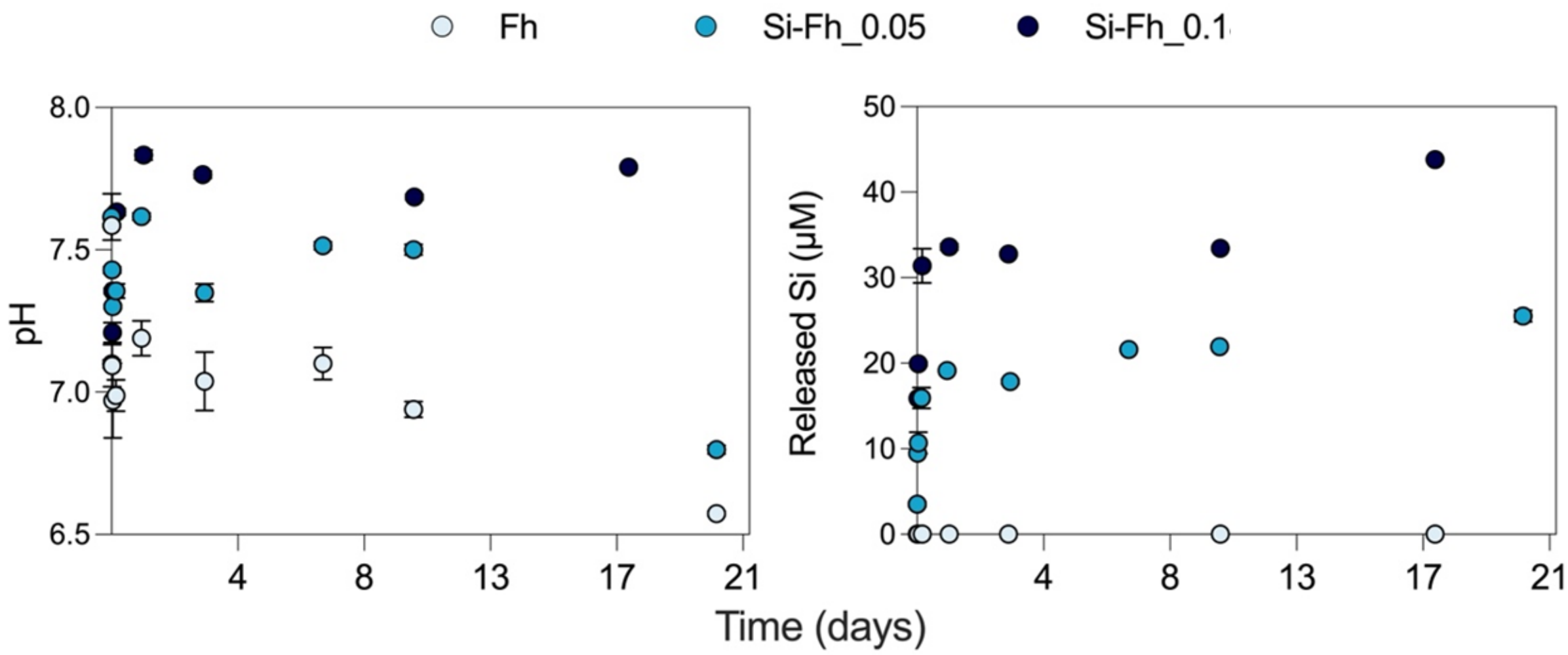

Figure S4. *Solution pH (left) and concentrations of dissolved Si (right) during the incubation of ferrihydrite (Fh) and Si–Fh coprecipitates (Si-Fh_0.05 and Si-Fh_0.1) with cysteine under anoxic conditions. Concentrations of dissolved Si in solution account for ~2-3% of the total Si content in the Si-Fh minerals, indicating minimal dissolution.*

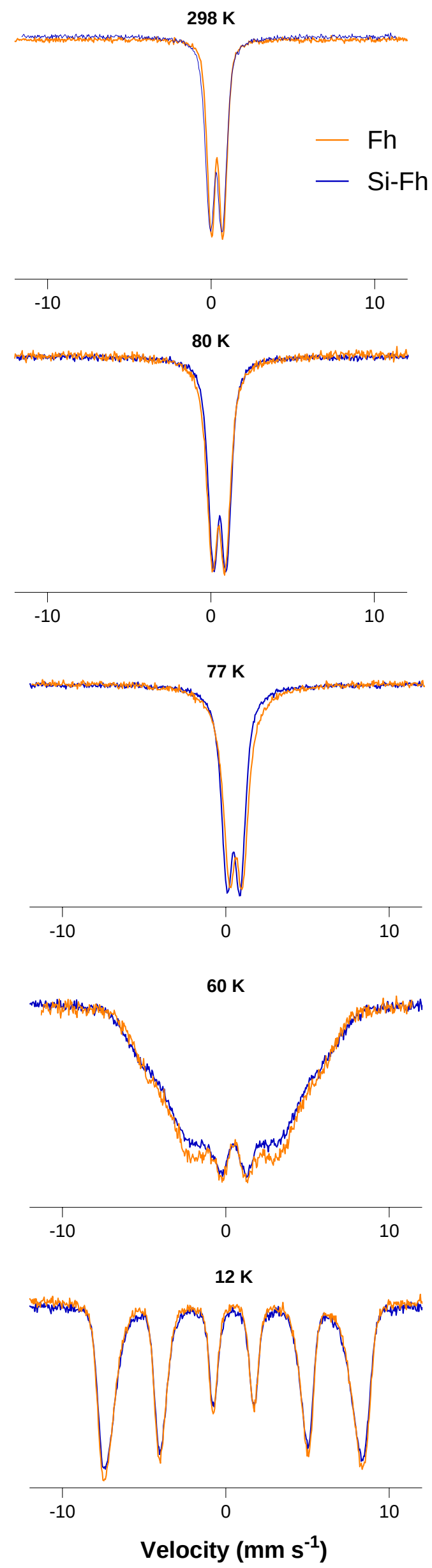

Figure S5. *Mössbauer spectra of untreated ferrihydrite (Fh) and Si–Fh_0.05 collected at 12 K, 60 K, 77 K, 80 K and 298K.*

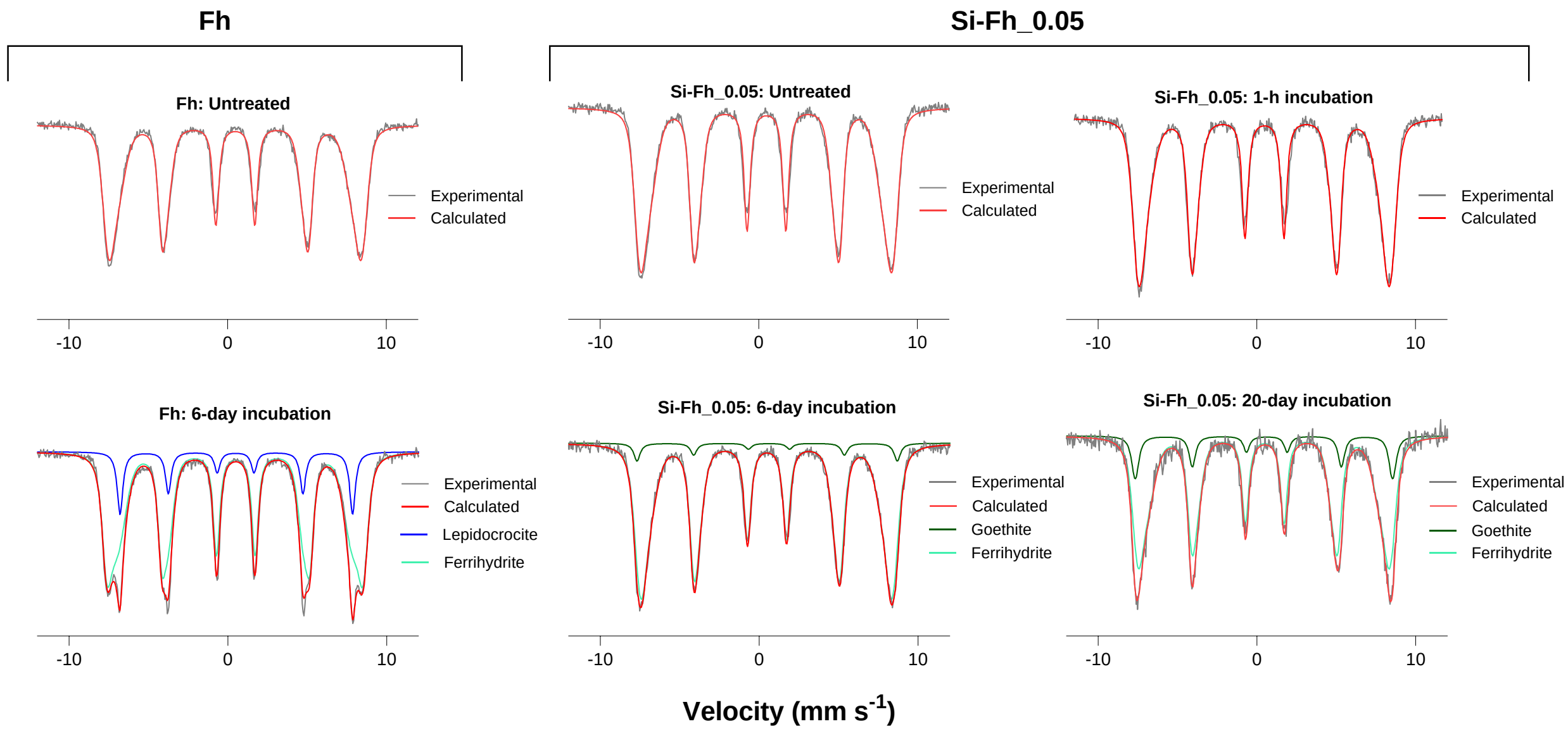

Figure S6. *Mössbauer spectra collected at 12 K for ferrihydrite (Fh) and Si–Fh_0.05 before (i.e., untreated) and after incubation with cysteine under anoxic conditions. Experimental data (gray) and calculated spectra (red) are shown, with spectral components assigned to Fh, goethite, and lepidocrocite as indicated. Incubation times are 1 h, 6 days, and 20 days.*

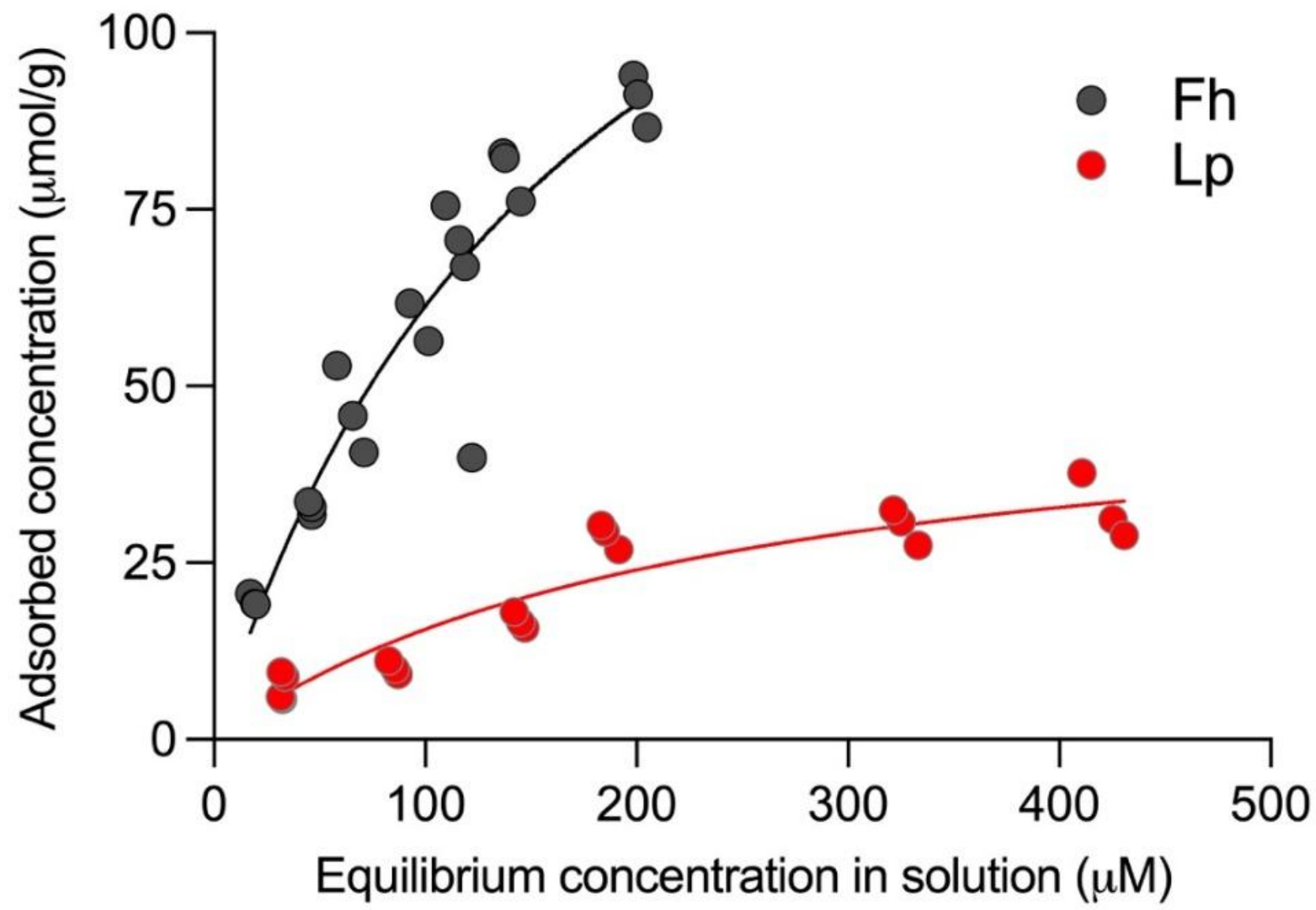

Figure S7. *Adsorption isotherms of cystine on ferrihydrite (Fh; black) and on lepidocrocite (Lp; red). The solid lines represent the non-linear regression fits of the Langmuir model. The calculated maximum adsorption and affinity parameters for Fh are:* q_{max}*=196.8±29.1 μmol/g,* K_L*=0.01935±0.00679 L/μmol,* R^2*=0.9579, and for Lp:* q_{max}*=52.02±17.14 μmol/g,* K_L*=0.00428±0.00268 L/μmol,* R^2*=0.8810.*

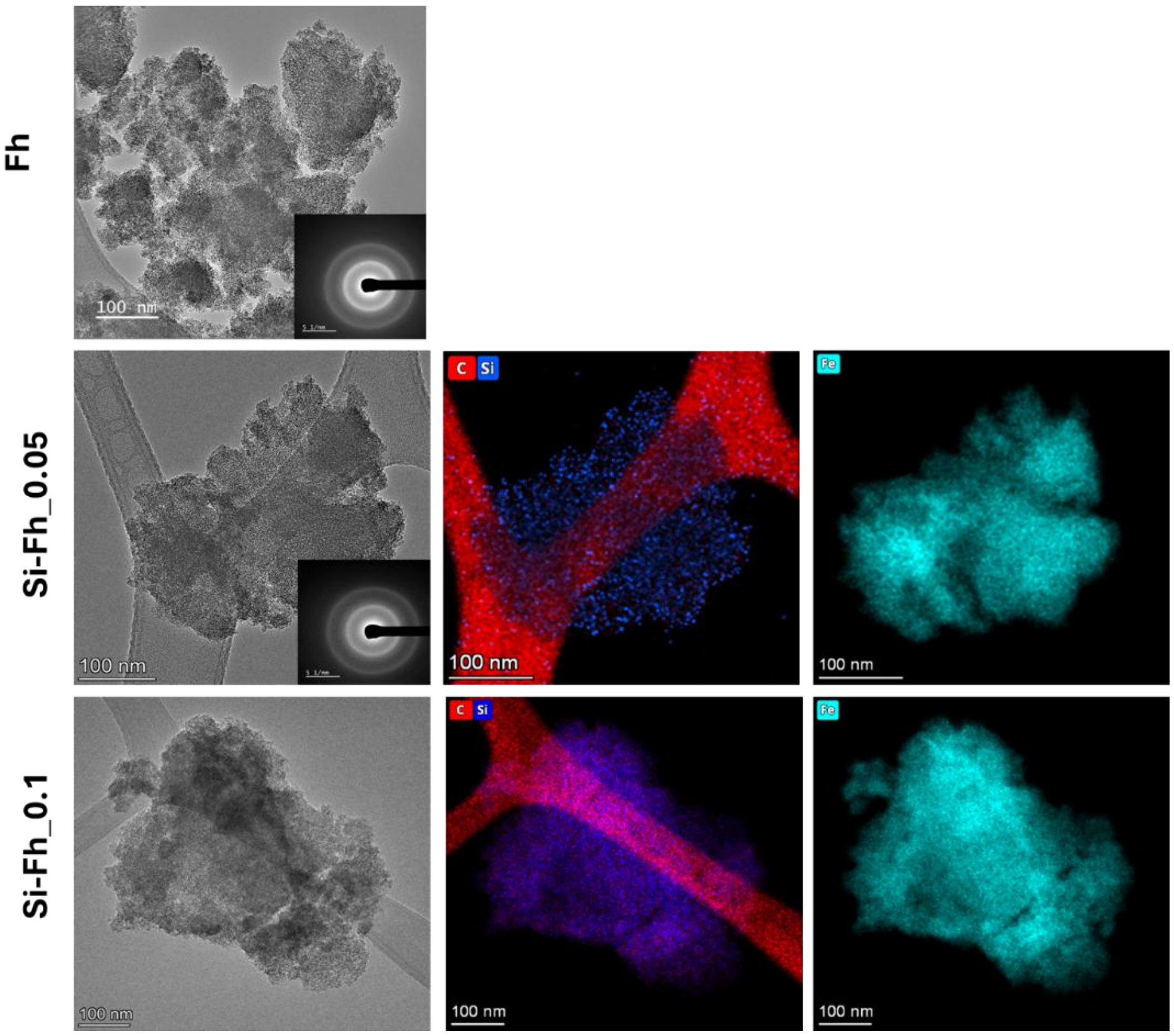

Figure S8. *TEM images of untreated ferrihydrite (Fh; top left), Si–Fh_0.05 (middle left) and Si-Fh_0.1 (bottom left), along with their corresponding electron diffraction rings in the bottom right corner. Corresponding EDX maps of C, Si, and Fe distribution in the Si-Fh_0.05 and Si-Fh_0.1 minerals are presented to their right (the C in red is attributed to the C-based grid). Si/Fe ratios in the minerals are presented in Table S1.*

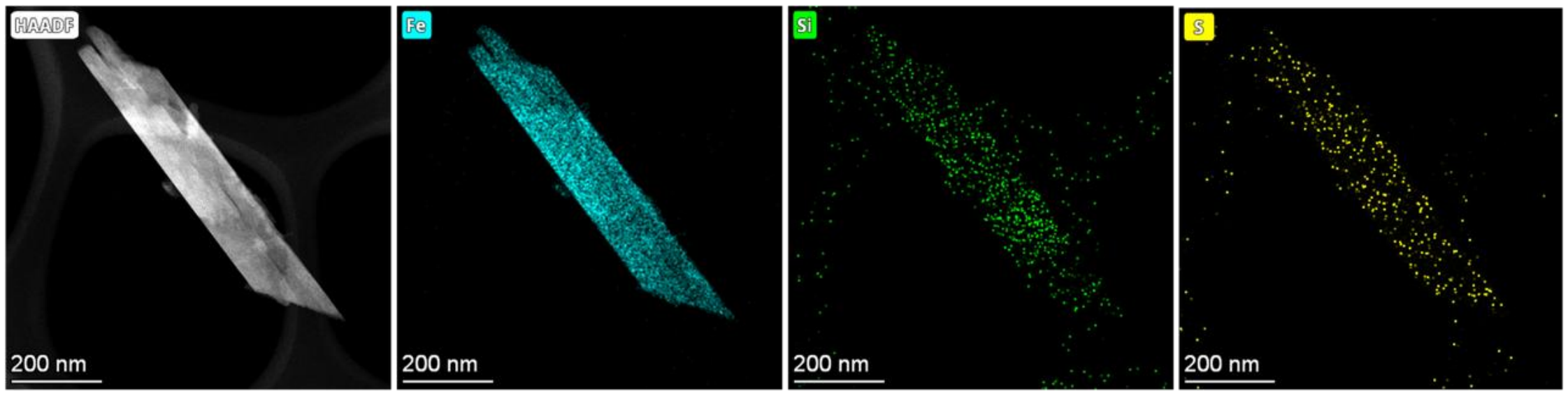

Figure S9. *TEM of a lepidocrocite (Lp) particle obtained after 20 days of incubation of Si-ferrihydrite (Si-Fh_0.05) with cysteine under anoxic conditions. Corresponding EDX maps show the distribution of Fe, Si, and S, indicating a uniform Fe framework with minor Si (Si/Fe=0.01) and negligible S association (S/Fe≈0).*

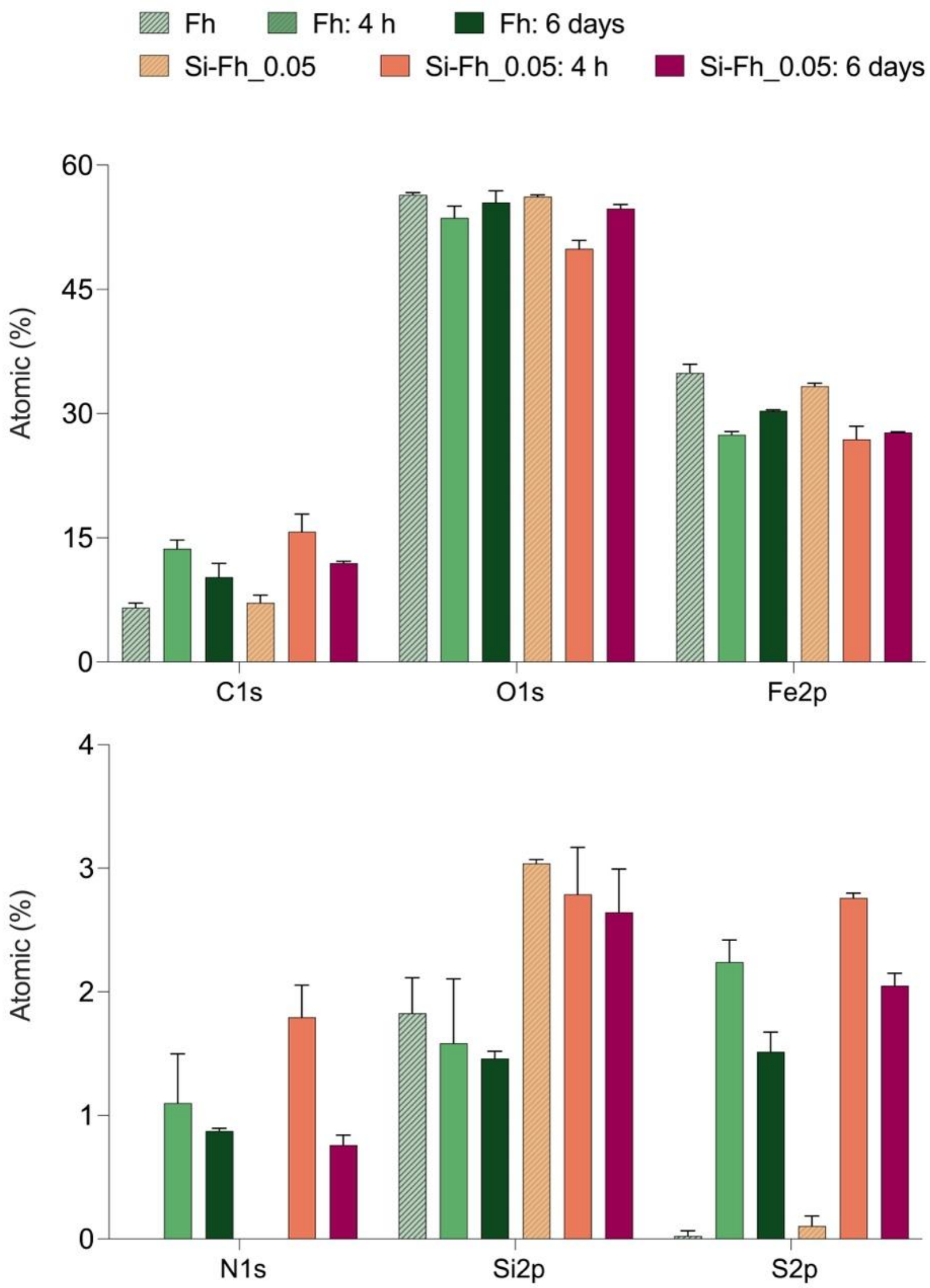

Figure S10. *Atomic percentages of surface elements (C 1s, O 1s, Fe 2p, N 1s, Si 2p, S 2p) measured by XPS for untreated ferrihydrite (Fh), Si–Fh_0.05, and the mineral products recovered after their incubation in the presence of cysteine under anoxic conditions for 4 h and 6 days.*

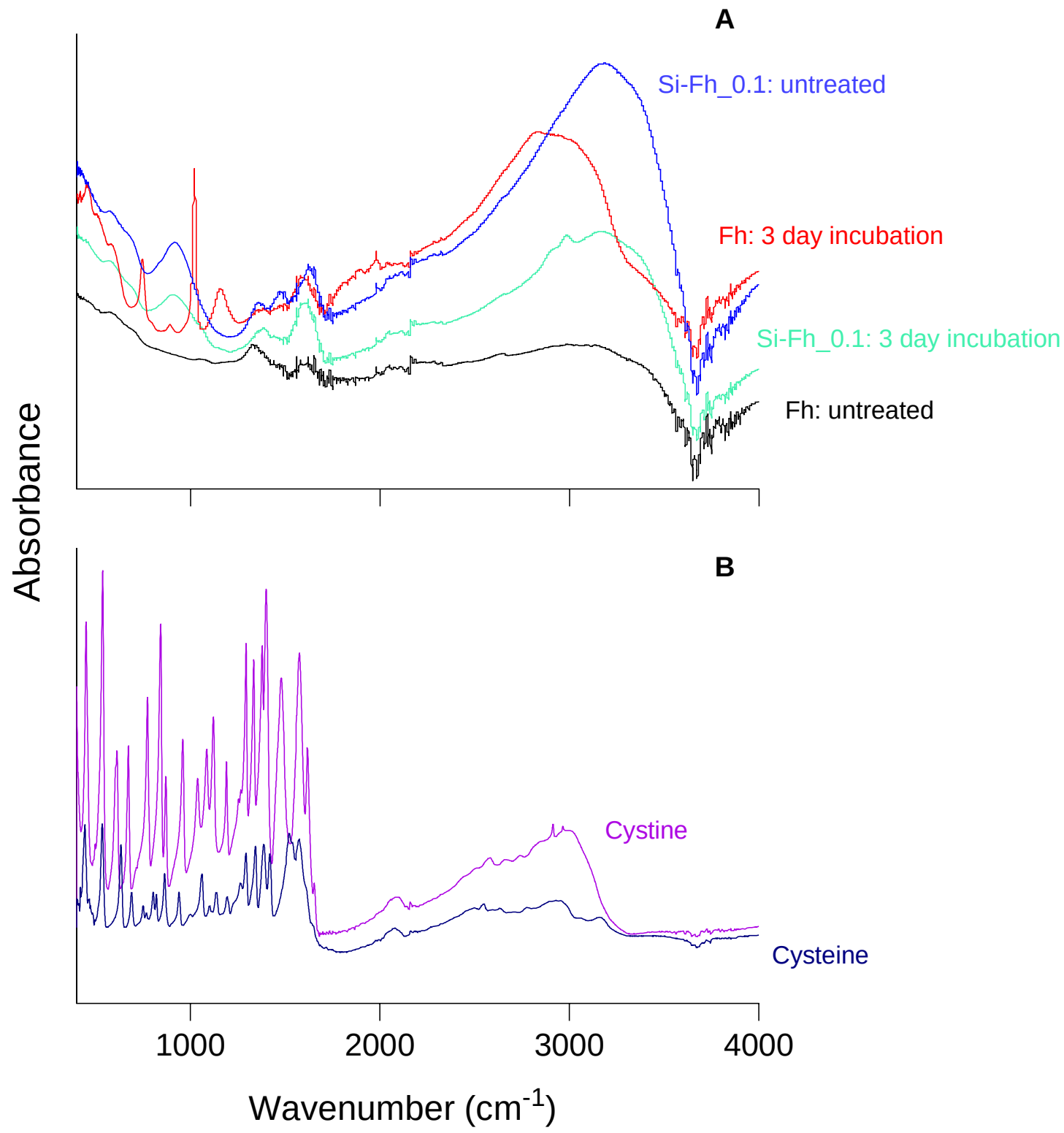

Figure S11. *FTIR spectra of (A) untreated ferrihydrite (Fh), untreated Si-Fh_0.1, and their respective samples following a 3-day incubation with cysteine under anoxic conditions (selected as example samples), and (B) cystine and cysteine standard powder samples.*

Bibliography

(1) Rietveld, H. M. Line Profiles of Neutron Powder-Diffraction Peaks for Structure Refinement. *Acta Crystallogr.* **1967**, *22* (1), 151–152. https://doi.org/10.1107/S0365110X67000234.

(2) Rietveld, H. M. A Profile Refinement Method for Nuclear and Magnetic Structures. *J. Appl. Crystallogr.* **1969**, *2* (2), 65–71. https://doi.org/10.1107/S0021889869006558.

(3) Rietveld, H. M. The Rietveld Method. *Phys. Scr.* **2014**, *89* (9), 098002. https://doi.org/10.1088/0031-8949/89/9/098002.

(4) ThomasArrigo, L. K.; Byrne, J. M.; Kappler, A.; Kretzschmar, R. Impact of Organic Matter on Iron(II)-Catalyzed Mineral Transformations in Ferrihydrite–Organic Matter Coprecipitates. *Environ. Sci. Technol.* **2018**, *52* (21), 12316–12326. https://doi.org/10.1021/acs.est.8b03206.

(5) Doelsch, E.; Stone, W. E. E.; Petit, S.; Masion, A.; Rose, J.; Bottero, J.-Y.; Nahon, D. Speciation and Crystal Chemistry of Fe(III) Chloride Hydrolyzed in the Presence of SiO_4 Ligands. 2. Characterization of Si−Fe Aggregates by FTIR and ^{29} Si Solid-State NMR. *Langmuir* **2001**, *17* (5), 1399–1405. https://doi.org/10.1021/la0013188.

(6) Schulz, K.; ThomasArrigo, L. K.; Kaegi, R.; Kretzschmar, R. Stabilization of Ferrihydrite and Lepidocrocite by Silicate during Fe(II)-Catalyzed Mineral Transformation: Impact on Particle Morphology and Silicate Distribution. *Environ. Sci. Technol.* **2022**, *56* (9), 5929–5938. https://doi.org/10.1021/acs.est.1c08789.

(7) Zachara, J. M.; Heald, S. M.; Jeon, B.-H.; Kukkadapu, R. K.; Liu, C.; McKinley, J. P.; Dohnalkova, A. C.; Moore, D. A. Reduction of Pertechnetate [Tc(VII)] by Aqueous Fe(II) and the Nature of Solid Phase Redox Products. *Geochim. Cosmochim. Acta* **2007**, *71* (9), 2137–2157. https://doi.org/10.1016/j.gca.2006.10.025.

(8) Schwertmann, U.; Cornell, R. M. *Iron Oxides in the Laboratory: Preparation and Characterization*; John Wiley & Sons, 2008.

(9) Rancourt, D. G.; Ping, J. Y. Voigt-Based Methods for Arbitrary-Shape Static Hyperfine Parameter Distributions in Mössbauer Spectroscopy. *Nucl. Inst Methods Phys. Res. B* **1991**, *58* (1), 85–97. https://doi.org/10.1016/0168-583X(91)95681-3.

(10) Coates, J. Interpretation of Infrared Spectra, A Practical Approach. In *Encyclopedia of Analytical Chemistry*; Meyers, R. A., Ed.; Wiley, 2000. https://doi.org/10.1002/9780470027318.a5606.
